# Impact of Post-Primary Treatment Hormone Replacement Therapy on Survival in Women with Cervical Cancer

**DOI:** 10.3390/cancers18172841

**Published:** 2026-09-02

**Authors:** Hee Joong Lee, Banghyun Lee

**Affiliations:** 1 Department of Obstetrics & Gynecology, Uijeongbu St. Mary’s Hospital, College of Medicine, The Catholic University of Korea, Seoul 11765, Republic of Korea; heejoong@catholic.ac.kr; 2 Department of Obstetrics and Gynecology, Inha University Hospital, College of Medicine, Inha University, Incheon 22332, Republic of Korea

**Keywords:** cervical cancer, hormone replacement therapy, overall survival

## Abstract

**Background/Objectives**: The effects of hormone replacement therapy (HRT) after primary treatment on survival outcomes in women with cervical cancer (CC) remain unclear. This nationwide cohort study evaluated the association between post-primary treatment HRT and overall survival (OS) in women with CC. **Methods**: We identified 15,261 women aged ≤60 years with CC who received primary treatment using the Korean Health Insurance Review and Assessment Service database. HRT users were defined as women who received HRT prescriptions for ≥28 days within one year after completion of primary treatment. Stabilized inverse probability of treatment weighting (IPTW) was used to reduce baseline differences between HRT users and non-users, followed by multivariable Cox regression to further adjust for potential confounding. Landmark analyses were performed to address potential immortal time bias. **Results**: Among 15,261 women, 4118 (27.0%) received HRT. In IPTW-weighted analyses, HRT use was significantly associated with improved OS in both multivariable models (adjusted HR, 0.544; 95% CI, 0.502–0.590; *p* < 0.001 and adjusted HR, 0.653; 95% CI, 0.574–0.742; *p* < 0.001, respectively). This association was generally maintained in landmark analyses at 1, 3, and 5 years after diagnosis and remained significant across localized, regional, and distant disease in stage-stratified analyses. The association was most consistently observed among women with squamous cell carcinoma. **Conclusions**: Post-primary treatment HRT was associated with improved OS in women with CC. Further prospective studies are needed to clarify the clinical implications of this association.

## Simple Summary

The effects of hormone replacement therapy (HRT) after cervical cancer (CC) treatment on survival remain unclear. Previous studies have suggested that HRT does not adversely affect survival in women with CC, but the available evidence has been limited by small sample sizes. This nationwide study included 15,261 women aged 60 years or younger with CC and examined whether HRT after primary treatment was associated with overall survival. HRT use was associated with improved overall survival, and this association generally remained after accounting for differences in patient and treatment characteristics and across several additional analyses. The association was observed across different stages of CC and was most consistent among women with squamous cell carcinoma. Although differences between HRT users and non-users may still have influenced the results, these findings provide additional evidence regarding the association between post-treatment HRT use and survival in women with CC.

## 1. Introduction

Cervical cancer (CC) is the fourth most common cancer and a leading cause of cancer-related death among women worldwide, accounting for a substantial global disease burden [1]. Globally, an estimated 662,044 cases of CC and 348,709 deaths were reported in 2022, according to the Global Cancer Observatory (GLOBOCAN) [2]. Although the incidence of CC has decreased steadily, upward trends have been observed among women aged <39 years [2,3,4]. Approximately 50% of CC cases occur in women aged <49 years, with peak incidence occurring among women aged 40–49 years, according to the Korea Central Cancer Registry [3]. Many women are diagnosed with CC before menopause and undergo iatrogenic menopause after cancer treatment [2,3,4]. Thus, hormone replacement therapy (HRT) is critical for relieving menopausal symptoms that can negatively affect quality of life, including vasomotor symptoms, genitourinary syndrome of menopause, and bone loss [5,6].

Only a few small studies have investigated the effects of HRT use after treatment for CC on survival outcomes [7,8,9,10,11,12]. In a randomized controlled trial (RCT) in which women with early-stage CC were treated with surgery or radiation (*n* = 120), no significant difference in the five-year overall survival (OS) or recurrence rate was observed between women receiving HRT and those receiving a placebo [9]. Similarly, in women with early-stage cervical adenocarcinoma (AC) treated with surgery or radiation, a retrospective study (*n* = 70) showed that HRT was not associated with survival outcomes, including progression-free survival (PFS) and OS [10]. Another retrospective study of women with early-stage AC treated with surgery or radiation (*n* = 33) reported that HRT was not associated with PFS or disease-specific survival (DSS), whereas OS was higher among HRT users [11]. In a retrospective study of women with CC treated with radiation (*n* = 293), no difference in recurrence-free survival (RFS) was observed between HRT users and non-users, whereas OS was higher among HRT users [12]. Nevertheless, the significance of these findings is limited by the small sample sizes of these studies.

Despite the absence of clear evidence indicating harm, the impact of post-primary treatment HRT on survival outcomes in women with CC has not been definitively established. The limited size and heterogeneity of previous studies restrict the strength of the available evidence. To address this gap, we conducted a nationwide cohort study using Korean population-based data to evaluate the association between post-primary treatment HRT and survival outcomes in women with CC.

## 2. Materials and Methods

A nationwide, population-based dataset was used for this study, derived from the Korean Health Insurance Review and Assessment Service (HIRA), which is integrated with the National Health Insurance system covering approximately 98% of the population [13,14]. This database has been linked to the national cancer registry, enabling comprehensive identification of cancer cases and longitudinal follow-up. Women diagnosed with CC based on pathology and corresponding diagnostic codes recorded in HIRA between 1 July 2007 and 31 December 2021 were included. Their healthcare records were tracked through 31 December 2023 to assess outcomes. All data were anonymized prior to analysis in accordance with the Bioethics and Safety Act of South Korea. Approval for this study was obtained from the Institutional Review Board of Inha University Hospital (No. 2024-01-025) on 22 January 2024, and the requirement for informed consent was waived due to the retrospective nature of the study.

Eligible patients were identified using administrative and diagnostic coding systems, including the International Classification of Diseases, 10th revision (ICD-10), the Korea Health Insurance Medical Care Expenses dataset (2017 and 2019 versions), and HIRA Drug Ingredient Codes. CC cases were defined as women with pathology-confirmed diagnoses recorded between 2007 and 2021, together with corresponding ICD-10 codes (C53). To ensure the inclusion of primary CC cases, individuals with any diagnostic codes for other malignancies within the two years preceding the CC diagnosis were excluded.

Patients were excluded if they were younger than 19 years or older than 60 years at the time of CC diagnosis, did not receive primary treatment, or received primary treatment outside the window of 60 days before or after the initial pathological diagnosis. Additional exclusion criteria included a history of venous thromboembolism (VTE) prior to primary treatment, use of HRT before primary treatment, initiation of HRT more than one year after completion of primary treatment, or insufficient HRT exposure (defined as <28 days of HRT use within the first year after completion of primary treatment combined with ≥28 days of HRT use initiated beyond one year). Patients with histologic types other than squamous cell carcinoma (SCC), AC, adenosquamous carcinoma (ASC), or neuroendocrine carcinoma (NC) were also excluded. HRT users (HRT group) were defined as patients who received HRT prescriptions for ≥28 days within one year after completion of primary treatment, whereas non-users (control group) were defined as those who did not receive HRT at any time during follow-up.

Low socioeconomic status (SES) was defined as Medicaid use. The Charlson Comorbidity Index (CCI) was calculated using data obtained during the 365 days preceding the initial diagnosis of CC [15]. Tumor characteristics included histologic subtypes (SCC, AC, ASC, and NC) and stage (localized, regional, distant, or unknown). Primary treatment categories included surgery alone, surgery + adjuvant radiotherapy, surgery + adjuvant chemotherapy, radiotherapy alone, radiotherapy + adjuvant chemotherapy, neoadjuvant chemotherapy + radiotherapy ± adjuvant chemotherapy, and chemotherapy alone. Neoadjuvant chemotherapy was defined as chemotherapy administered during the 12 weeks preceding primary radiotherapy. Adjuvant treatment was defined as chemotherapy or radiotherapy administered within 12 weeks after primary surgery, or chemotherapy administered within 12 weeks after primary radiotherapy. Primary treatment modalities, including surgery, radiotherapy, and chemotherapy, were identified based on claims records within 60 days before or after the date of the initial pathological diagnosis of CC. Surgical procedures included total hysterectomy, radical hysterectomy, trachelectomy (radical or simple), and conization, confirmed by concurrent pathology records. When additional surgery was performed after conization, patients were reclassified according to the subsequent surgical procedure. Radiotherapy included external beam radiation therapy (EBRT) and vaginal brachytherapy (VB). Concurrent chemoradiotherapy (CCRT) was defined as the combined use of radiotherapy (EBRT or VB) and chemotherapy with a concurrent diagnostic code for CC. Chemotherapy exposure was identified using prescription records for cytotoxic or targeted agents (carboplatin, cisplatin, docetaxel, fluorouracil, gemcitabine, ifosfamide, irinotecan, mitomycin, paclitaxel, topotecan, and bevacizumab) administered with a concurrent CC diagnosis code. Hormone therapy was defined as prescriptions for estrogen-only therapy (ET), estrogen–progesterone therapy (EPT), or tibolone, accompanied by a concurrent CC diagnosis code. Postoperative venous thromboembolism (VTE) was defined as at least two prescriptions for anticoagulants in conjunction with diagnostic codes for deep vein thrombosis, pulmonary embolism, or both, after primary treatment. Prophylactic anticoagulant use was defined as at least two prescriptions for anticoagulants without accompanying VTE diagnostic codes after primary treatment. Anticoagulants included unfractionated heparin, low-molecular-weight heparin, fondaparinux, warfarin, and direct oral anticoagulants. OS was defined as the interval from the date of initial pathological diagnosis of CC to death or the end of follow-up. Death was identified using the HIRA death code or the predefined operational definition. Women without claim records for more than one year were classified as probable deaths according to the predefined operational definition, and their dates of death were imputed by adding a randomly assigned interval of 1–365 days to the last recorded healthcare encounter.

### Statistical Analyses

All statistical analyses were conducted using SAS version 9.4 (SAS Institute Inc., Cary, NC, USA). Continuous variables were compared using Student’s *t*-test, whereas categorical variables were compared using the chi-square test or Fisher’s exact test, as appropriate. Associations between clinical variables and OS, as well as between HRT duration and OS, were assessed using Cox proportional hazards regression models in univariable and multivariable analyses. Hazard ratios (HRs) and 95% confidence intervals (CIs) were estimated using Cox regression.

Because several treatment-related variables were highly correlated, two separate multivariable models were constructed to minimize potential multicollinearity. The same predefined covariate sets were consistently used, as applicable, across the multivariable Cox regression analyses. Multivariable model I was adjusted for age, SES, CCI, year of cancer diagnosis, stage, primary treatment, postoperative VTE, and prophylactic anticoagulant use, whereas multivariable model II was adjusted for age, SES, CCI, year of cancer diagnosis, stage, type of surgery, chemotherapy regimen, postoperative VTE, and prophylactic anticoagulant use. For analyses according to HRT duration, multivariable models III and IV used the same covariate sets as models I and II, respectively, with HRT use replaced by HRT duration categories and no HRT use used as the reference group. For covariate categories with sparse events, estimates affected by quasi-complete separation were reported as not estimable (NE), as specified in the corresponding table footnotes.

There were no missing data for the variables included in the analyses; therefore, no imputation was performed for missing covariate data. The linearity assumption for age was assessed using restricted cubic spline functions within the Cox models. The median age (47 years) was used as the reference value, and non-linearity was evaluated using Wald tests for the spline terms. The proportional hazards assumption was graphically assessed for the 1-, 3-, and 5-year landmark Cox models using standardized cumulative score process plots with simulated paths (Figure S1).

Stabilized inverse probability of treatment weighting (IPTW) was applied to reduce baseline differences between HRT users and non-users. Covariate balance after weighting was assessed using absolute standardized mean differences (SMDs), with values >0.1 indicating residual imbalance. The IPTW-weighted cohort was subsequently analyzed using Cox regression with the same predefined covariate sets as those used in multivariable models I and II.

Stage- and histology-stratified Cox regression analyses were additionally performed in the original cohort. Stage-stratified analyses were performed separately for localized, regional, and distant disease. Patients with unknown stage were excluded from these analyses because they could not be assigned to a clinically interpretable stage category. Histology-stratified analyses were performed separately for SCC, AC, ASC, and NC.

To evaluate the potential impact of the operational definition of death, a sensitivity analysis was performed in which patients with imputed death dates (i.e., those without healthcare claims for more than one year whose death dates had been operationally assigned) were instead treated as right-censored at their last recorded healthcare encounter. The Cox models were then repeated using only deaths identified by the HIRA death code as events.

To address potential immortal time bias associated with HRT exposure, a time-dependent Cox model was additionally performed, with HRT use treated as a time-varying covariate according to the actual prescription period. Person-time during HRT use was classified as exposed, whereas person-time before HRT initiation and after HRT discontinuation was classified as unexposed. In addition, landmark analyses were performed at 1, 3, and 5 years after the date of CC diagnosis. For each landmark analysis, only women who survived to the corresponding landmark were included, and HRT exposure before each landmark was determined using the same predefined HRT exposure criteria as in the primary analysis. HRT duration accumulated before each landmark was also evaluated in additional sensitivity analyses.

All statistical tests were two-sided, and a *p* value < 0.05 was considered statistically significant.

## 3. Results

**Figure 1:**
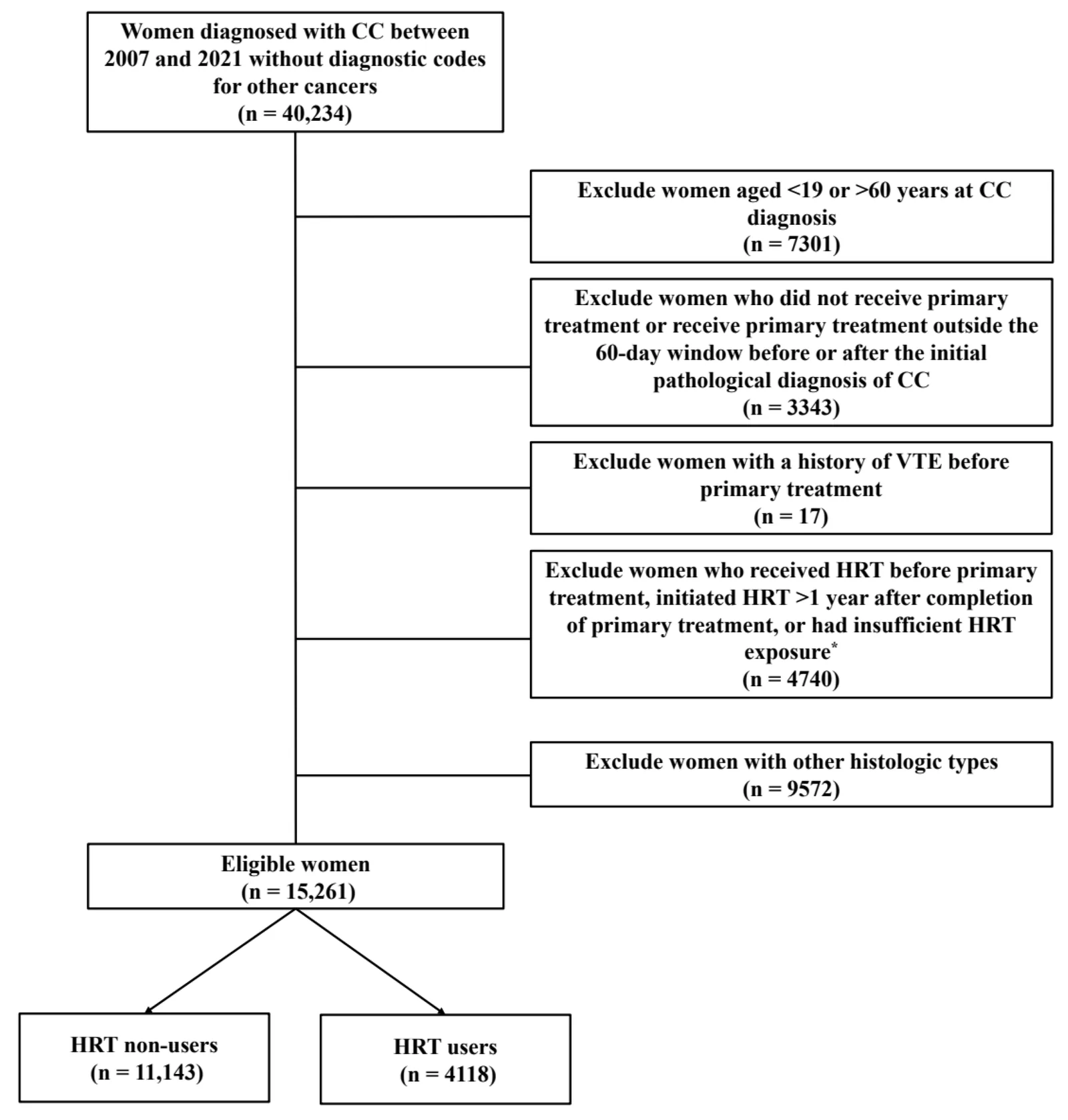
Flowchart of study population selection. CC, cervical cancer; HRT, hormone replacement therapy; VTE, venous thromboembolism. * Insufficient HRT exposure was defined as <28 days of HRT use within the first year after completion of primary treatment combined with ≥28 days of HRT use initiated beyond one year.

Initially, 40,234 women diagnosed with CC between 2007 and 2021 were identified from the Korean cancer registration data linked to the HIRA database. Of these, 15,261 women met the study eligibility criteria (Figure 1), including 4118 (27.0%) in the HRT group and 11,143 (73.0%) in the control group.

### 3.1. Characteristics According to HRT Use in Women with CC

Table 1 presents the baseline characteristics of the study population. The mean age was 46.2 ± 8.6 years overall, 45.2 ± 7.6 years in the HRT group, and 46.6 ± 9.0 years in the control group. HRT was initiated at a mean of 0.8 ± 1.5 years after completion of primary treatment, and the mean duration of HRT was 3.0 ± 3.3 years. After stabilized IPTW, most baseline covariates were well balanced between the HRT and non-HRT groups; however, residual imbalance remained in several variables, particularly primary treatment categories (Table 2). These variables were therefore additionally adjusted for in the IPTW-weighted multivariable Cox models (Table 3).

### 3.2. OS and Risk Factors in Women with CC

In the IPTW-weighted Cox analysis, HRT use was significantly associated with improved OS in multivariable analysis I (HR, 0.544; 95% CI, 0.502–0.590; *p* < 0.001). Increasing age was also associated with improved OS. Women with mid- or high SES showed significantly improved OS compared with those with low SES. Compared with women with localized disease, OS was significantly worse in those with regional or distant disease. Compared with surgery alone as the primary treatment, all other primary treatment categories were associated with worse OS. Postoperative VTE and prophylactic anticoagulant use were also associated with worse OS. CCI was not significantly associated with OS. Compared with 2007, all subsequent years of cancer diagnosis were associated with worse OS (Table 3).

In multivariable analysis II, HRT use remained significantly associated with improved OS (HR, 0.653; 95% CI, 0.574–0.742; *p* < 0.001). Other variables, including age, SES, stage, postoperative VTE, and prophylactic anticoagulant use, showed results consistent with those observed in multivariable analysis I. Compared with platinum-based chemotherapy, treatment with bevacizumab ± any agents was associated with worse OS, whereas other agents showed no significant difference. Compared with a CCI of 0, a CCI of 1 was associated with improved OS, whereas the other CCI categories were not significantly associated with OS. Compared with total hysterectomy, conization was associated with improved OS, whereas the other surgical approaches showed no significant difference. Compared with 2007, cancer diagnoses in 2013, 2020, and 2021 were associated with worse OS (Table 3).

The conventional Cox analysis in the original cohort showed results generally consistent with those of the IPTW-weighted analysis (Table S1). HRT use was significantly associated with improved OS in both multivariable analysis I (HR, 0.525; 95% CI, 0.484–0.570; *p* < 0.001) and multivariable analysis II (HR, 0.578; 95% CI, 0.507–0.658; *p* < 0.001). The associations of other major clinical factors with OS were also generally consistent with those observed in the IPTW-weighted analysis.

Restricted cubic spline analyses for age are shown in Figure S2. Significant non-linearity was observed in the univariable analysis (*p* for non-linearity = 0.046) and multivariable analysis I (*p* for non-linearity < 0.001), whereas no significant non-linearity was observed in multivariable analysis II (*p* for non-linearity = 0.176).

### 3.3. OS According to HRT Use in Women with CC

The mean follow-up duration for the overall cohort was 6.8 ± 4.4 years (7.9 ± 4.3 years in the HRT group and 6.4 ± 4.3 years in the control group). The median follow-up duration was 8.0 years (interquartile range [IQR], 4.1–11.6 years) in the HRT group and 5.7 years (IQR, 2.5–10.0 years) in the control group.

Deaths were identified using both the HIRA death code and the operational definition. A total of 3734 deaths (24.5%) were identified. A total of 772 women in the HRT group (18.8%) and 2962 women in the control group (26.6%) died during follow-up, with maximum follow-up periods of 15.9 and 16.4 years, respectively. Within 10 years, 674 women (16.4%) in the HRT group and 2693 women (24.2%) in the control group died, whereas within 5 years, 476 women (11.6%) and 2174 women (19.5%) died, respectively. The 10-year OS rates were 79.6% in the HRT group and 69.1% in the control group (95% CI, 78.1–80.9% and 68.0–70.1%, respectively). The corresponding 5-year OS rates were 87.1% and 77.6% (95% CI, 86.0–88.1% and 76.7–78.4%, respectively).

The HIRA death code identified 2633 deaths (17.3%) during follow-up, including 543 (13.2%) in the HRT group and 2090 (18.8%) in the control group. To assess the impact of the operational definition used for patients without healthcare claims for more than one year, we performed a sensitivity analysis in which patients with imputed death dates were treated as right-censored at their last recorded healthcare encounter. The association between HRT use and improved OS remained consistent with the primary analysis (multivariable analysis I: adjusted HR, 0.578; 95% CI, 0.524–0.637; *p* < 0.001; multivariable analysis II: adjusted HR, 0.551; 95% CI, 0.473–0.641; *p* < 0.001; Table S2).

### 3.4. Sensitivity Analysis Using Landmark Cox Regression

Table S3 presents the baseline characteristics of the study population at the 1-, 3-, and 5-year landmarks. At the 1-year landmark, 14,343 women were included, comprising 3252 (22.7%) in the HRT group and 11,091 (77.3%) in the control group. At the 3-year landmark, 11,246 women were included, comprising 3170 (28.2%) and 8076 (71.8%) women in the HRT and control groups, respectively. At the 5-year landmark, 8998 women were included, comprising 2735 (30.4%) and 6263 (69.6%) women in the HRT and control groups, respectively.

In the time-dependent Cox analysis, no deaths occurred during HRT-exposed person-time, resulting in separation, and the estimated HR approached zero. Consequently, this analysis was considered statistically non-informative and was not interpreted as evidence of an association between HRT exposure and mortality. Landmark analyses performed at 1, 3, and 5 years after diagnosis were therefore used as the main sensitivity analyses to address potential immortal time bias. HRT use remained significantly associated with improved OS in both multivariable analyses at the 1-year landmark (multivariable analysis I: adjusted HR, 0.655; 95% CI, 0.600–0.715; *p* < 0.001; multivariable analysis II: adjusted HR, 0.629; 95% CI, 0.549–0.719; *p* < 0.001) and the 3-year landmark (multivariable analysis I: adjusted HR, 0.749; 95% CI, 0.671–0.835; *p* < 0.001; multivariable analysis II: adjusted HR, 0.753; 95% CI, 0.633–0.896; *p* = 0.001). At the 5-year landmark, the association remained significant in multivariable analysis I (adjusted HR, 0.734; 95% CI, 0.639–0.844; *p* < 0.001), whereas it was attenuated and did not reach statistical significance in multivariable analysis II (adjusted HR, 0.814; 95% CI, 0.643–1.030; *p* = 0.086) (Table 4).

### 3.5. OS According to HRT Duration Using Landmark Cox Regression

In additional sensitivity analyses incorporating HRT duration before each landmark, HRT durations of up to 1 year were generally associated with improved subsequent OS. HRT use for ≤0.5 years was significantly associated with improved OS in both multivariable analyses III and IV at the 1- and 3-year landmarks (adjusted HRs, 0.652–0.781) and in multivariable analysis III at the 5-year landmark (adjusted HR, 0.717). HRT use for 0.5–1 year was consistently associated with improved OS in both multivariable analyses III and IV at all three landmarks (adjusted HRs, 0.587–0.740). In contrast, HRT use for longer than 1 year was not significantly associated with subsequent OS at the 3- and 5-year landmarks (Table S4).

### 3.6. Sensitivity Analysis Using Stage-Stratified Cox Regression

In stage-stratified sensitivity analyses, HRT use remained significantly associated with improved OS across all stage groups. Among women with localized disease, the adjusted HRs for HRT use were 0.614 (95% CI, 0.530–0.712; *p* < 0.001) in multivariable analysis I and 0.669 (95% CI, 0.529–0.845; *p* < 0.001) in multivariable analysis II. Similar associations were observed among women with regional disease (multivariable analysis I: adjusted HR, 0.514; 95% CI, 0.455–0.581; *p* < 0.001; multivariable analysis II: adjusted HR, 0.532; 95% CI, 0.444–0.637; *p* < 0.001) and distant disease (multivariable analysis I: adjusted HR, 0.431; 95% CI, 0.353–0.526; *p* < 0.001; multivariable analysis II: adjusted HR, 0.531; 95% CI, 0.357–0.792; *p* = 0.002) (Table 5).

### 3.7. Sensitivity Analysis Using Histology-Stratified Cox Regression

In histology-stratified sensitivity analyses, HRT use was significantly associated with improved OS among women with SCC in both multivariable analyses (multivariable analysis I: adjusted HR, 0.522; 95% CI, 0.477–0.571; *p* < 0.001; multivariable analysis II: adjusted HR, 0.571; 95% CI, 0.493–0.662; *p* < 0.001). Among women with AC, HRT use was significantly associated with improved OS in multivariable analysis I (adjusted HR, 0.517; 95% CI, 0.392–0.683; *p* < 0.001), but not in multivariable analysis II. Similarly, among women with ASC, HRT use was significantly associated with improved OS in multivariable analysis I (adjusted HR, 0.626; 95% CI, 0.414–0.947; *p* = 0.027), but not in multivariable analysis II. No significant association between HRT use and OS was observed among women with NC in either multivariable analysis (Table S5).

## 4. Discussion

In the present study, HRT use was significantly associated with improved OS compared with non-use. This association remained significant after stabilized IPTW and was generally maintained in landmark analyses at 1, 3, and 5 years after diagnosis, although it was attenuated and did not reach statistical significance in multivariable analysis II at the 5-year landmark. Stage-stratified analyses demonstrated a significant association between HRT use and improved OS across localized, regional, and distant disease. In histology-stratified analyses, the association was consistently observed among women with SCC in both multivariable analyses and among women with AC and ASC in multivariable analysis I, whereas no significant association was observed among women with NC. In additional landmark analyses incorporating HRT duration before each landmark, HRT durations of up to 1 year were generally associated with improved subsequent OS, whereas longer durations were not consistently associated with OS. Overall, the association between HRT use and improved OS remained generally consistent across the primary and sensitivity analyses addressing baseline differences, potential immortal time bias, stage heterogeneity, histologic subgroups, and uncertainty related to death ascertainment.

A previous retrospective study (*n* = 1019) reported a mean age at CC diagnosis of 47.1 ± 14.4 years [16]. Current guidelines recommend initiating HRT in women younger than 60 years of age or within 10 years of menopause, considering the balance between benefits and potential risks [5,6,8]. HRT may improve menopausal symptoms and quality of life; however, depending on the type and duration of HRT and individual patient risk factors, HRT may be associated with an increased risk of conditions such as cardiovascular disease, stroke, and venous thromboembolism [5,6,8]. Therefore, the potential benefits and risks of HRT should be carefully balanced in women with CC. Accordingly, only women aged ≤60 years were included in the present study. Despite this age restriction, the mean ages of women in the HRT and control groups were comparable to the mean age reported in the previous study [16].

Post-primary treatment HRT might be associated with a potential survival benefit in women with CC, but the evidence remains insufficient despite previous studies reporting no adverse impact on survival [7,8,9,10,11,12]. An RCT and a retrospective study conducted in Poland and South Korea, respectively, showed similar OS in women with and without HRT after primary treatment (surgery or radiation), whereas two retrospective studies conducted in the United Kingdom and the Netherlands reported higher OS among HRT users than among non-users [9,10,11,12]. Moreover, the five-year recurrence rate in the RCT, PFS in two retrospective studies, DSS in one retrospective study, and RFS in another retrospective study were similar in HRT users and non-users after primary treatment [9,10,11,12]. These findings suggest that HRT use may not adversely affect survival and may potentially be associated with improved survival across different populations and healthcare settings. Nevertheless, the available evidence is derived from only a few small studies and requires confirmation in larger studies [9,10,11,12]. Thus, this large-scale nationwide cohort study demonstrated a significant association between HRT use and improved OS. In the IPTW-weighted analyses, HRT use remained significantly associated with improved OS after multivariable adjustment (Table 3). The maximum follow-up periods were 15.9 and 16.4 years in the HRT and control groups, respectively.

Disease stage is an important prognostic factor in CC and is closely related to the selection of primary treatment. To address heterogeneity related to disease extent and treatment selection, stage-stratified analyses were performed. The association between HRT use and improved OS remained significant in both multivariable analyses across localized, regional, and distant disease, suggesting that the observed association was not restricted to a particular stage group (Table 5). Treatment-related findings, however, should be interpreted cautiously. In particular, conization was associated with improved OS compared with total hysterectomy in the IPTW-weighted multivariable analysis II, whereas this association was not observed in the conventional multivariable analysis of the original cohort or within the stage-stratified analyses. These differences may reflect changes in covariate distribution after weighting as well as residual treatment-selection confounding. Because conization is generally performed in selected women with earlier and more favorable disease, the observed association should not be interpreted as evidence of a direct survival benefit of conization.

SCC is the most common histologic subtype of CC, accounting for 80–90% of cases, whereas AC accounts for 10–20% [7,17,18]. Although SCC is not considered a hormone-dependent carcinoma, estrogen receptor (ER) and progesterone receptor (PR) are expressed in SCC, and previous studies have reported a reduced risk of SCC in women treated with HRT [7,18,19,20,21,22]. ER and PR are also expressed in approximately one-third of AC cases. Moreover, previous studies have reported a slightly increased risk of AC in women treated with HRT, suggesting that this association might be related to the histologic similarity between the endocervical glandular epithelium and the endometrium [7,20,21,22,23,24]. In a retrospective study (*n* = 39), however, ER and PR expression in CC was not correlated with clinicopathological parameters, such as tumor size, grade, or lymph node status, and was not associated with OS or disease-free survival [24]. Moreover, two small retrospective studies reported that HRT use after primary treatment was not associated with PFS, DSS, or OS in women with early-stage AC [10,11]. Currently, HRT after CC treatment is not considered contraindicated in CC survivors, including those with AC [7,17,18,25]. However, the safety of HRT in women with AC remains somewhat unclear. Therefore, large-scale studies evaluating the safety and potential impact of post-treatment HRT on survival outcomes according to the histologic subtype of CC are warranted. In the present large cohort study, HRT use was significantly associated with improved OS among women with SCC in both multivariable analyses and among women with AC and ASC in multivariable analysis I, whereas no significant association was observed among women with NC (Table S5). In the histology-stratified analyses, HRT use was associated with an approximately 42.9–47.8% lower risk of death among women with SCC; in multivariable analysis I, the corresponding risk reductions were approximately 48.3% and 37.4% among women with AC and ASC, respectively (Table S5).

The impact of the duration of HRT use on survival outcomes in women with CC has not been reported. A few studies have reported inconclusive results regarding the incidence of CC according to HRT duration [20,21,22,26]. A retrospective nationwide cohort study (*n* = 1784) reported that among women with ovarian cancer receiving postoperative HRT, OS was significantly higher in women who received HRT for >5 years than in those who received HRT for ≤0.5 years (unadjusted HR, 0.234; 95% CI, 0.059–0.936; *p* = 0.040) [27]. Importantly, in the present study, HRT use remained significantly associated with improved OS in both multivariable analyses at the 1- and 3-year landmarks and in multivariable analysis I at the 5-year landmark. However, the association was attenuated and no longer statistically significant in multivariable analysis II at the 5-year landmark (Table 4). This attenuation should be considered in the context of the reduced sample size at the 5-year landmark and the predominantly short duration of HRT exposure in this cohort. Nevertheless, the non-significant result in multivariable analysis II at the 5-year landmark tempers the strength of evidence for a sustained long-term association between HRT use and improved OS and warrants cautious interpretation of the overall findings. Sensitivity analyses incorporating HRT duration before each landmark showed that HRT durations of up to 1 year were generally associated with improved subsequent OS, whereas HRT use for longer than 1 year was not significantly associated with subsequent OS at the 3- and 5-year landmarks (Table S4). However, these findings for longer HRT duration should be interpreted cautiously because the longer-duration groups included fewer patients and events, resulting in less precise estimates. In particular, quasi-complete separation occurred in the 4–5-year HRT duration category at the 5-year landmark, precluding reliable estimation of the adjusted HR. Therefore, the lack of statistically significant associations for longer HRT durations should not be interpreted as evidence of the absence of a survival association.

Only a minority of women with perimenopause or menopause after CC treatment receive HRT. In a retrospective cohort study (*n* = 1826), among women aged <50 years at CC diagnosis who experienced treatment-induced iatrogenic menopause, 39.0% received HRT, and the median duration of HRT use was only 60 days [28]. Another retrospective study (*n* = 202) reported that among women with iatrogenic menopause due to CC treatment, 48.0% received counseling, an HRT prescription, or both, and the median duration of HRT use was 37.3 months (IQR, 17.5–65.8) [29]. Consistent with these previous studies, only 27.0% of women with CC in the present study received HRT, with a mean duration of HRT use of 3.0 ± 3.3 years. In addition, women diagnosed with CC between 2007 and 2011 were more frequently represented in the HRT group, whereas those diagnosed between 2016 and 2021 were less frequently represented, with no significant differences between the groups for 2012–2015, suggesting lower HRT use among women diagnosed in more recent years in this study population (Table 1). Among HRT users included in the landmark analyses, HRT exposure was also generally of short duration: HRT durations of ≤1 year accounted for 100%, 90.0%, and 87.5% of HRT users at the 1-, 3-, and 5-year landmarks, respectively (Table S3). These findings indicate that both the proportion of women receiving HRT and the duration of HRT use after CC treatment were relatively limited in this cohort. Given that HRT use was associated with improved OS in the present study and that previous studies have reported no adverse impact on survival, these findings highlight a clinically important gap in survivorship care, in which HRT use remains uncommon despite the lack of clear evidence of adverse survival effects. Further prospective studies are needed to clarify the role of HRT in survivorship care after CC treatment.

A large nationwide cohort study reported that among women with CC (*n* = 49,514), the incidence of VTE after primary treatment was higher in women who received prophylactic anticoagulants than in those who did not (8.3% vs. 0.1%) [30]. In the present study, OS was lower in women who developed postoperative VTE and in those who received prophylactic anticoagulants. Given that both the previous study and the present study were based on the HIRA database and thus likely represented similar patient populations and clinical practice patterns, the association between prophylactic anticoagulant use and worse OS may reflect the higher underlying risk of postoperative VTE among women receiving prophylactic anticoagulants rather than an adverse effect of anticoagulant use itself (Table 3 and Table 4; Table S1).

Although HIRA contains death information, not all deaths are completely ascertained through claims-based records. Therefore, we initially used an operational definition to identify probable deaths among patients without healthcare claims for more than one year. To address concerns regarding potential bias introduced by this approach, we performed a sensitivity analysis in which these patients were treated as right-censored at their last recorded healthcare encounter and only deaths identified by the HIRA death code were considered events. The association between HRT use and improved OS remained materially unchanged in this sensitivity analysis.

This study represents the first large-scale analysis to evaluate the association between post-primary treatment HRT and survival outcomes in women with CC. In addition, it provides histology-specific and duration-specific analyses of survival outcomes. Several limitations should be considered. First, because this analysis was based on claims data, diseases other than CC and treatment exposures were identified indirectly from diagnostic and prescription codes without medical record review, which may have resulted in some degree of misclassification. Second, detailed treatment sequencing and timing following primary treatment could not be fully evaluated because of heterogeneous treatment patterns in this large nationwide cohort and limitations inherent to claims-based data. Third, prognostic factors such as detailed tumor stage, grade, performance status, recurrence, and lifestyle factors were not available in the HIRA dataset linked to cancer registry data. In addition, stage information was available only as SEER summary stage categories (localized, regional, and distant disease), which may have reduced staging precision compared with detailed International Federation of Gynecology and Obstetrics (FIGO) staging. Importantly, these unavailable or imprecisely measured clinical factors may have influenced both the likelihood of receiving HRT and survival outcomes, thereby contributing to residual or unmeasured confounding. Therefore, these unavailable or imprecisely measured clinical variables may have influenced the observed association between HRT use and improved OS. Nevertheless, age, SES, CCI, histologic type, SEER summary stage, primary treatment variables, postoperative VTE, and prophylactic anticoagulant use were available and incorporated into the analyses. Fourth, because the claims-based dataset did not include information regarding disease progression, recurrence, or cause of death, PFS, DSS, and RFS could not be evaluated, and potential competing risks related to non-cancer mortality could not be assessed. Therefore, the observed association with OS cannot be interpreted as evidence of improved CC-specific survival or better oncologic disease control. Because premature or early menopause may be associated with adverse cardiovascular consequences [31,32], the observed association between HRT use and improved OS could partly reflect non-cancer effects of HRT, including potential cardiovascular effects, rather than a direct effect on cancer outcomes. However, cardiovascular outcomes were not evaluated in the present study, and cause-specific mortality was not available; therefore, this possibility could not be assessed. The lack of information on disease progression and recurrence may also have contributed to residual or unmeasured confounding and influenced the observed association between HRT use and improved OS. However, we attempted to account for measured confounding using multivariable adjustment. Two separate multivariable models were constructed to minimize potential multicollinearity among treatment-related variables, and the association between HRT use and improved OS remained consistent across both models. Fifth, death ascertainment was subject to limitations inherent to the HIRA claims database. Because not all deaths were captured by the HIRA death code, an operational definition was used to identify probable deaths among patients without healthcare claims for more than one year, and their death dates were imputed. This approach may have introduced misclassification of death status or survival time. Nevertheless, the association between HRT use and improved OS remained materially unchanged in a sensitivity analysis in which these patients were treated as right-censored at their last recorded healthcare encounter and only deaths identified by the HIRA death code were considered events. Sixth, the definition of HRT exposure based on treatment initiation after primary treatment may have introduced immortal time bias. We therefore performed a time-dependent Cox analysis and landmark analyses at 1, 3, and 5 years after diagnosis. Although the time-dependent Cox model could not provide a reliable estimate because no deaths occurred during HRT-exposed person-time, the association between HRT use and improved OS was generally maintained in the landmark analyses, except in multivariable analysis II at the 5-year landmark. Nevertheless, these approaches cannot completely eliminate potential time-related biases inherent to the observational study design. Seventh, because many women changed hormone regimens repeatedly during follow-up, overlapping treatment exposures were common, and formulation-specific subgroup analyses according to ET, EPT, or tibolone could not be reliably performed. In addition, detailed evaluations according to HRT dose, route of administration, regimen, and treatment adherence could not be performed because of the heterogeneous patterns of HRT use and limitations inherent to claims-based data. Accordingly, the observed association represents overall HRT exposure and should not be interpreted as a formulation-specific effect. Eighth, this study included only women from South Korea; therefore, the findings may have limited generalizability to populations with different healthcare systems, demographic characteristics, HRT prescribing practices, and CC treatment patterns. External validation in other populations and healthcare settings is therefore required. Finally, despite the use of stabilized IPTW, multivariable adjustment, and multiple sensitivity analyses, residual and unmeasured confounding cannot be completely excluded. Although IPTW improved the balance of most measured baseline characteristics, some residual imbalance remained, particularly in primary treatment categories. Moreover, healthy user bias may have contributed to the observed association between HRT use and improved OS, as HRT users may differ from non-users in unmeasured characteristics, such as overall health status, healthcare utilization, health-seeking behavior, follow-up intensity, socioeconomic circumstances, or access to healthcare, that could not be adequately captured in the claims database. Given the magnitude of this association, it remains uncertain whether the effect size reflects a direct biological or therapeutic effect of HRT or is partly attributable to residual or unmeasured confounding and other biases inherent to this observational study. Stage-stratified analyses were performed to further address heterogeneity related to disease extent and treatment selection, and HRT use remained significantly associated with improved OS across localized, regional, and distant disease. However, these analytical approaches cannot fully account for residual and unmeasured differences between HRT users and non-users. Therefore, the observed association between HRT use and improved OS should not be interpreted as establishing a causal relationship.

## 5. Conclusions

This retrospective, nationwide, population-based cohort study showed that post-primary treatment HRT was associated with improved OS in women with CC. This association remained generally consistent after stabilized IPTW and across multiple sensitivity analyses and was most consistently observed among women with SCC. However, the attenuation of the association at the 5-year landmark and the methodological limitations inherent to this observational study warrant cautious interpretation of the findings and preclude establishing a causal relationship. Large-scale prospective studies or RCTs are warranted to further evaluate the association between post-primary treatment HRT and survival outcomes in women with CC.

## Figures and Tables

**Table 1:** Baseline characteristics of the original cohort according to HRT use.

	**No. (%)**			
**Characteristics**	**Total**	**HRT (−)**	**HRT (+)**	***p* Value**
No. of women	15,261 (100)	11,143 (73)	4118 (27)	
Age (years), mean (SD)	46.2 ± 8.6	46.6 ± 9.0	45.2 ± 7.6	<0.001
SES				
Low SES	2192 (14.4)	1530 (13.7)	662 (16.1)	<0.001
Mid- or high-SES	13,069 (85.6)	9613 (86.3)	3456 (83.9)	
CCI				
0	10,532 (69.0)	7683 (68.9)	2849 (69.2)	0.845
1	2036 (13.3)	1475 (13.2)	561 (13.6)	
2	1838 (12.0)	1350 (12.1)	488 (11.9)	
3	526 (3.4)	388 (3.5)	138 (3.4)	
≥4	329 (2.2)	247 (2.2)	82 (2.0)	
Year of cancer diagnosis				
2007	1124 (7.4)	710 (6.4)	414 (10.1)	<0.001
2008	1212 (7.9)	820 (7.4)	392 (9.5)	<0.001
2009	1027 (6.7)	698 (6.3)	329 (8.0)	<0.001
2010	1100 (7.2)	759 (6.8)	341 (8.3)	0.002
2011	1067 (7.0)	747 (6.7)	320 (7.8)	0.022
2012	1094 (7.2)	772 (6.9)	322 (7.8)	0.058
2013	1141 (7.5)	831 (7.5)	310 (7.5)	0.883
2014	1053 (6.9)	756 (6.8)	297 (7.2)	0.355
2015	1034 (6.8)	769 (6.9)	265 (6.4)	0.309
2016	1039 (6.8)	788 (7.1)	251 (6.1)	0.034
2017	950 (6.2)	745 (6.7)	205 (5.0)	<0.001
2018	941 (6.2)	754 (6.8)	187 (4.5)	<0.001
2019	854 (5.6)	663 (5.9)	191 (4.6)	0.002
2020	796 (5.2)	648 (5.8)	148 (3.6)	<0.001
2021	829 (5.4)	683 (6.1)	146 (3.5)	<0.001
Histologic types				
SCC	12,940 (84.8)	9448 (84.8)	3492 (84.8)	0.616
AC	1574 (10.3)	1158 (10.4)	416 (10.1)	
ASC	592 (3.9)	419 (3.8)	173 (4.2)	
Large cell NC	34 (0.2)	25 (0.2)	9 (0.2)	
Small cell NC	121 (0.8)	93 (0.8)	28 (0.7)	
Stage				
Localized	7785 (51.0)	5748 (51.6)	2037 (49.5)	0.020
Regional	5388 (35.3)	3751 (33.7)	1637 (39.8)	<0.001
Distant	1299 (8.5)	1047 (9.4)	252 (6.1)	<0.001
Unknown	789 (5.2)	597 (5.4)	192 (4.7)	0.085
Primary treatment				
Surgery alone	6261 (41.0)	5035 (45.2)	1226 (29.8)	<0.001
Surgery + adjuvant radiotherapy	3324 (21.8)	2109 (18.9)	1215 (29.5)	<0.001
Surgery + adjuvant chemotherapy	481 (3.2)	313 (2.8)	168 (4.1)	<0.001
Radiotherapy alone	527 (3.5)	390 (3.5)	137 (3.3)	0.603
Radiotherapy + adjuvant chemotherapy	1451 (9.5)	936 (8.4)	515 (12.5)	<0.001
Neoadjuvant chemotherapy + radiotherapy ± adjuvant chemotherapy	2036 (13.3)	1434 (12.9)	602 (14.6)	<0.001
Chemotherapy alone	1181 (7.7)	926 (8.3)	255 (6.2)	<0.001
Surgery				
Total hysterectomy	478 (3.1)	374 (3.4)	104 (2.5)	0.009
Radical hysterectomy	6525 (42.8)	4560 (40.9)	1965 (47.7)	<0.001
Trachelectomy (radical or simple)	150 (1.0)	121 (1.1)	29 (0.7)	0.034
Conization	2913 (19.1)	2402 (21.6)	511 (12.4)	<0.001
Radiotherapy				
EBRT ± VB	2083 (13.6)	1405 (12.6)	678 (16.5)	<0.001
VB alone	1769 (11.6)	1266 (11.4)	503 (12.2)	0.144
CCRT	162 (1.1)	89 (0.8)	73 (1.8)	<0.001
Chemotherapy				
Platinum-based chemotherapy	897 (5.9)	666 (6.0)	231 (5.6)	0.392
Other agents	4 (0.0)	4 (0.0)	0 (0.0)	0.224 ^a^
Bevacizumab ± any agents	280 (1.8)	256 (2.3)	24 (0.6)	<0.001
Postoperative VTE				
(−)	14,960 (98.0)	10,900 (97.8)	4060 (98.6)	0.002
(+)	301 (2.0)	243 (2.2)	58 (1.4)	
Prophylactic anticoagulants				
(−)	8505 (55.7)	6260 (56.2)	2245 (54.5)	0.067
(+)	6756 (44.3)	4883 (43.8)	1873 (45.5)	
**HRT-related characteristics among HRT users only** (***n* = 4118**) **^b^**				
**Characteristics**			**HRT** (**+**)	
HRT formulation ^c^				
ET			2005 (48.7)	
EPT			2766 (67.2)	
Tibolone			1352 (32.8)	
Duration of HRT (years)				
≤0.5			2338 (56.8)	
0.5–1			1182 (28.7)	
1–2			254 (6.2)	
2–3			129 (3.1)	
3–4			50 (1.2)	
4–5			46 (1.1)	
>5			119 (2.9)	
Time from primary treatment completion to HRT initiation (years), mean (SD)			0.8 ± 1.5	

Abbreviations: AC, cervical adenocarcinoma; ASC, adenosquamous carcinoma; CCI, Charlson comorbidity index; CCRT, concurrent chemoradiotherapy; EBRT, external beam radiation therapy; ET, estrogen-only therapy; EPT, estrogen–progesterone therapy; HRT, hormone replacement therapy; NC, neuroendocrine carcinoma; No, number; SES, socioeconomic status; SCC, squamous cell carcinoma; SD, standard deviation; VB, vaginal brachytherapy; VTE, venous thromboembolism. ^a^ Fisher’s exact test was used for this analysis. ^b^ HRT-related characteristics are presented descriptively only for women who received HRT and were not included in the baseline balance comparison between the HRT and non-HRT groups. ^c^ The HRT formulation categories were not mutually exclusive because some women received more than one HRT formulation during follow-up.

**Table 2:** Baseline characteristics after stabilized IPTW in the weighted pseudo-population.

	No. (%)			
Characteristics	Total	HRT (−)	HRT (+)	SMD ^a^
No. of women	15,246 (100)	11,131 (73)	4115 (27)	
Age (years), mean (SD)	46.3 ± 8.6	46.2 ± 9.0	46.3 ± 7.4	0.011
SES				
Low SES	2184 (14.3)	1597 (14.3)	662 (16.1)	0.050
Mid- or high-SES	13,062 (85.7)	9534 (85.7)	3456 (83.9)	
CCI				
0	10,518 (69.0)	7691 (69.1)	2849 (69.2)	0.002
1	2023 (13.3)	1457 (13.1)	561 (13.6)	0.015
2	1843 (12.1)	1353 (12.2)	488 (11.9)	0.009
3	528 (3.5)	387 (3.5)	138 (3.4)	0.005
≥4	334 (2.2)	243 (2.2)	82 (2.0)	0.014
Year of cancer diagnosis				
2007	1148 (7.5)	735 (6.6)	414 (10.1)	**0.127**
2008	1219 (8.0)	844 (7.6)	392 (9.5)	0.068
2009	1037 (6.8)	717 (6.4)	329 (8.0)	0.062
2010	1102 (7.2)	772 (6.9)	341 (8.3)	0.053
2011	1064 (7.0)	756 (6.8)	320 (7.8)	0.038
2012	1095 (7.2)	785 (7.1)	322 (7.8)	0.027
2013	1147 (7.5)	843 (7.6)	310 (7.5)	0.004
2014	1043 (6.8)	757 (6.8)	297 (7.2)	0.016
2015	1036 (6.8)	763 (6.9)	265 (6.4)	0.020
2016	1038 (6.8)	774 (7.0)	251 (6.1)	0.036
2017	940 (6.2)	721 (6.5)	205 (5.0)	0.064
2018	927 (6.1)	728 (6.5)	187 (4.5)	0.088
2019	840 (5.5)	640 (5.7)	191 (4.6)	0.050
2020	783 (5.1)	631 (5.7)	148 (3.6)	0.100
2021	826 (5.4)	666 (6.0)	146 (3.5)	**0.118**
Histologic types				
SCC	12,891 (84.6)	9416 (84.6)	3492 (84.8)	0.006
AC	1600 (10.5)	1148 (10.3)	416 (10.1)	0.007
ASC	591 (3.9)	432 (3.9)	173 (4.2)	0.015
Large cell NC	37 (0.2)	29 (0.3)	9 (0.2)	0.020
Small cell NC	127 (0.8)	105 (0.9)	28 (0.7)	0.022
Stage				
Localized	7841 (51.4)	5690 (51.1)	2037 (49.5)	0.032
Regional	5357 (35.1)	3921 (35.2)	1637 (39.8)	0.095
Distant	1265 (8.3)	946 (8.5)	252 (6.1)	0.092
Unknown	783 (5.1)	574 (5.2)	192 (4.7)	0.023
Primary treatments				
Surgery alone	6387 (41.9)	4596 (41.3)	1226 (29.8)	**0.242**
Surgery + adjuvant radiotherapy	3311 (21.7)	2425 (21.8)	1215 (29.5)	**0.177**
Surgery + adjuvant chemotherapy	486 (3.2)	353 (3.2)	168 (4.1)	0.048
Radiotherapy alone	513 (3.4)	382 (3.4)	137 (3.3)	0.006
Radiotherapy + adjuvant chemotherapy	1428 (9.4)	1047 (9.4)	515 (12.5)	0.099
Neoadjuvant chemotherapy + radiotherapy ± adjuvant chemotherapy	1986 (13.0)	1469 (13.2)	602 (14.6)	0.040
Chemotherapy alone	1136 (7.4)	858 (7.7)	255 (6.2)	0.059
Surgery				
Total hysterectomy	484 (3.2)	350 (3.1)	104 (2.5)	0.036
Radical hysterectomy	6610 (43.4)	4785 (43.0)	1965 (47.7)	0.095
Trachelectomy (radical or simple)	147 (1.0)	109 (1.0)	29 (0.7)	0.033
Conization	2942 (19.3)	2130 (19.1)	511 (12.4)	**0.185**
Radiotherapy				
EBRT ± VB	2041 (13.4)	1504 (13.5)	678 (16.5)	0.084
VB alone	1725 (11.3)	1278 (11.5)	503 (12.2)	0.022
CCRT	161 (1.1)	116 (1.0)	73 (1.8)	0.068
Chemotherapy				
Platinum-based chemotherapy	878 (5.8)	651 (5.8)	231 (5.6)	0.009
Other agents ^b^	3 (0.0)	3 (0.0)	0 (0.0)	0.000
Bevacizumab ± any agents	255 (1.7)	204 (1.8)	24 (0.6)	**0.110**
Postoperative VTE				
(−)	14,939 (98.0)	10,885 (97.8)	4060 (98.6)	0.060
(+)	307 (2.0)	246 (2.2)	58 (1.4)	
Prophylactic anticoagulants				
(−)	8483 (55.6)	6225 (55.9)	2245 (54.5)	0.028
(+)	6763 (44.4)	4906 (44.1)	1873 (45.5)	

Values after IPTW are presented as weighted means or proportions; No denotes the weighted sample size. Abbreviations: AC, cervical adenocarcinoma; ASC, adenosquamous carcinoma; CCI, Charlson comorbidity index; CCRT, concurrent chemoradiotherapy; EBRT, external beam radiation therapy; HRT, hormone replacement therapy; IPTW, inverse probability of treatment weighting; No, number; SES, socioeconomic status; SCC, squamous cell carcinoma; SD, standard deviation; SMD, standardized mean difference; VB, vaginal brachytherapy; VTE, venous thromboembolism. ^a^ Absolute SMD values > 0.1, indicating residual imbalance, are shown in bold. ^b^ SMD could not be computed by the standard formula for this category because the proportion was 0% in both the HRT (−) and HRT (+) groups (division by zero); by convention, the SMD between two identical proportions of 0% is reported as 0.000, indicating no imbalance between groups.

**Table 3:** IPTW-weighted Cox proportional hazards models for OS.

	Univariable Analysis		Multivariable Analysis I		Multivariable Analysis II	
Variables	HR (95% CI)	*p* Value	HR (95% CI)	*p* Value	HR (95% CI)	*p* Value
Age (years)	1.006 (1.003–1.010)	0.001	0.988 (0.981–0.994)	<0.001	0.984 (0.980–0.988)	<0.001
SES						
Low SES	Ref		Ref		Ref	
Mid- or high-SES	0.659 (0.608–0.714)	<0.001	0.844 (0.730–0.975)	0.022	0.846 (0.779–0.918)	<0.001
CCI						
0	Ref		Ref		Ref	
1	0.869 (0.785–0.962)	0.007	0.921 (0.781–1.085)	0.324	0.880 (0.794–0.975)	0.015
2	0.956 (0.865–1.057)	0.378	0.850 (0.708–1.021)	0.082	0.914 (0.827–1.011)	0.081
3	0.921 (0.768–1.103)	0.371	0.805 (0.569–1.140)	0.222	0.859 (0.716–1.031)	0.103
≥4	1.184 (0.962–1.458)	0.110	1.317 (0.878–1.975)	0.184	1.154 (0.936–1.423)	0.180
Year of cancer diagnosis						
2007	Ref		Ref		Ref	
2008	1.268 (1.089–1.476)	0.002	1.234 (1.060–1.436)	0.007	1.051 (0.816–1.355)	0.699
2009	1.326 (1.125–1.562)	<0.001	1.190 (1.011–1.402)	0.037	1.023 (0.780–1.342)	0.867
2010	1.359 (1.152–1.602)	<0.001	1.228 (1.041–1.449)	0.015	0.945 (0.719–1.244)	0.689
2011	1.456 (1.231–1.722)	<0.001	1.357 (1.147–1.605)	<0.001	1.104 (0.835–1.458)	0.488
2012	1.598 (1.353–1.888)	<0.001	1.331 (1.126–1.574)	<0.001	1.078 (0.819–1.418)	0.594
2013	1.875 (1.593–2.208)	<0.001	1.610 (1.366–1.897)	<0.001	1.356 (1.041–1.766)	0.024
2014	1.628 (1.367–1.939)	<0.001	1.336 (1.120–1.594)	<0.001	0.889 (0.667–1.186)	0.425
2015	1.729 (1.450–2.062)	<0.001	1.398 (1.169–1.671)	<0.001	0.765 (0.564–1.038)	0.088
2016	1.752 (1.464–2.097)	<0.001	1.399 (1.165–1.680)	<0.001	0.748 (0.550–1.016)	0.064
2017	1.748 (1.443–2.118)	<0.001	1.343 (1.105–1.632)	<0.001	0.792 (0.570–1.101)	0.166
2018	1.964 (1.612–2.392)	<0.001	1.340 (1.095–1.640)	<0.001	0.720 (0.507–1.024)	0.068
2019	3.058 (2.497–3.745)	<0.001	2.172 (1.768–2.669)	<0.001	1.163 (0.802–1.687)	0.427
2020	6.541 (5.229–8.183)	<0.001	3.422 (2.727–4.294)	<0.001	1.716 (1.147–2.568)	0.009
2021	23.970 (18.790–30.600)	<0.001	7.131 (5.554–9.156)	<0.001	4.874 (3.045–7.802)	<0.001
Stage						
Localized	Ref		Ref		Ref	
Regional	2.711 (2.509–2.929)	<0.001	1.583 (1.390–1.802)	<0.001	1.955 (1.791–2.133)	<0.001
Distant	9.237 (8.429–10.120)	<0.001	3.690 (3.067–4.439)	<0.001	4.907 (4.414–5.454)	<0.001
Unknown	1.836 (1.580–2.134)	<0.001	1.577 (1.153–2.156)	0.004	1.702 (1.461–1.982)	<0.001
Primary treatments						
Surgery alone	Ref		Ref			
Surgery + adjuvant radiotherapy	2.030 (1.841–2.239)	<0.001	1.374 (1.236–1.528)	<0.001		
Surgery + adjuvant chemotherapy	1.751 (1.454–2.109)	<0.001	1.443 (1.194–1.744)	<0.001		
Radiotherapy alone	4.728 (4.109–5.440)	<0.001	3.135 (2.703–3.636)	<0.001		
Radiotherapy + adjuvant chemotherapy	3.255 (2.931–3.616)	<0.001	2.272 (2.017–2.559)	<0.001		
Neoadjuvant chemotherapy + radiotherapy ± adjuvant chemotherapy	3.260 (2.931–3.626)	<0.001	2.128 (1.889–2.398)	<0.001		
Chemotherapy alone	7.387 (6.626–8.235)	<0.001	3.292 (2.905–3.730)	<0.001		
Surgery						
Total hysterectomy	Ref				Ref	
Radical hysterectomy	0.630 (0.507–0.782)	<0.001			0.783 (0.607–1.010)	0.060
Trachelectomy (radical or simple)	0.667 (0.430–1.035)	0.071			1.131 (0.662–1.930)	0.653
Conization	0.386 (0.305–0.488)	<0.001			0.750 (0.563–0.999)	0.049
Radiotherapy						
EBRT ± VB	Ref					
VB alone	1.048 (0.954–1.151)	0.331				
CCRT	1.065 (0.856–1.325)	0.574				
Chemotherapy						
Platinum-based chemotherapy	Ref				Ref	
Other agents	1.113 (0.614–2.019)	0.724			1.262 (0.587–2.714)	0.551
Bevacizumab ± any agents	4.260 (3.878–4.679)	<0.001			3.611 (2.948–4.424)	<0.001
Postoperative VTE						
(−)	Ref		Ref		Ref	
(+)	2.903 (2.499–3.371)	<0.001	1.213 (1.039–1.416)	0.014	1.311 (1.005–1.711)	0.046
Prophylactic anticoagulants						
(−)	Ref		Ref		Ref	
(+)	2.353 (2.202–2.514)	<0.001	2.179 (2.034–2.335)	<0.001	2.341 (2.056–2.665)	<0.001
HRT						
(−)	Ref		Ref		Ref	
(+)	0.571 (0.527–0.619)	<0.001	0.544 (0.502–0.590)	<0.001	0.653 (0.574–0.742)	<0.001

Abbreviations: CCI, Charlson comorbidity index; CCRT, concurrent chemoradiotherapy; CI, confidence interval; EBRT, external beam radiation therapy; HR, hazard ratio; HRT, hormone replacement therapy; OS, overall survival; SES, socioeconomic status; VB, vaginal brachytherapy; VTE, venous thromboembolism. Multivariable analyses I and II were adjusted using the same predefined covariate sets as those used throughout the study.

**Table 4:** Sensitivity analysis using landmark Cox regression at the 1-, 3-, and 5-year landmarks.

	1 Year				3 Year				5 Year			
	Multivariable Analysis I		Multivariable Analysis II		Multivariable Analysis I		Multivariable Analysis II		Multivariable Analysis I		Multivariable Analysis II	
Variables	HR (95% CI)	*p* Value	HR (95% CI)	*p* Value	HR (95% CI)	*p* Value	HR (95% CI)	*p* Value	HR (95% CI)	*p* Value	HR (95% CI)	*p* Value
Age (years)	0.987 (0.983–0.991)	<0.001	0.988 (0.981–0.994)	<0.001	0.994 (0.989–1.000)	0.047	1.000 (0.990–1.010)	0.967	0.998 (0.991–1.005)	0.599	1.006 (0.993–1.020)	0.374
SES												
Low SES	Ref		Ref		Ref		Ref		Ref		Ref	
Mid- or high-SES	0.875 (0.803–0.952)	0.002	0.855 (0.735–0.993)	0.041	0.878 (0.777–0.993)	0.038	0.845 (0.685–1.042)	0.115	0.909 (0.771–1.071)	0.255	0.759 (0.571–1.007)	0.056
CCI												
0	Ref		Ref		Ref		Ref		Ref		Ref	
1	0.909 (0.819–1.008)	0.071	0.969 (0.821–1.144)	0.714	0.897 (0.776–1.037)	0.142	1.038 (0.827–1.303)	0.746	0.768 (0.628–0.938)	0.010	0.877 (0.626–1.229)	0.445
2	0.935 (0.843–1.037)	0.204	0.872 (0.722–1.054)	0.157	0.929 (0.802–1.075)	0.321	0.891 (0.683–1.164)	0.399	0.875 (0.724–1.056)	0.164	0.833 (0.575–1.207)	0.334
3	0.898 (0.747–1.079)	0.252	0.856 (0.604–1.215)	0.385	1.009 (0.788–1.292)	0.941	0.798 (0.496–1.283)	0.351	0.992 (0.715–1.377)	0.964	0.899 (0.475–1.702)	0.745
≥4	1.184 (0.953–1.471)	0.126	1.339 (0.881–2.035)	0.172	1.083 (0.789–1.488)	0.621	1.620 (0.928–2.827)	0.090	1.293 (0.888–1.884)	0.180	1.857 (0.950–3.630)	0.070
Year of cancer diagnosis												
2007	Ref		Ref		Ref		Ref		Ref		Ref	
2008	1.219 (1.040–1.428)	0.014	1.061 (0.813–1.384)	0.665	1.606 (1.289–2.002)	<0.001	1.286 (0.883–1.871)	0.190	2.241 (1.686–2.978)	<0.001	1.410 (0.850–2.338)	0.183
2009	1.235 (1.042–1.464)	0.015	1.103 (0.833–1.460)	0.493	1.747 (1.368–2.232)	<0.001	1.546 (1.038–2.303)	0.032	3.014 (2.178–4.173)	<0.001	2.354 (1.371–4.043)	0.002
2010	1.300 (1.095–1.544)	0.003	1.017 (0.765–1.353)	0.906	2.306 (1.806–2.946)	<0.001	1.654 (1.107–2.471)	0.014	4.939 (3.543–6.885)	<0.001	2.730 (1.556–4.790)	<0.001
2011	1.418 (1.191–1.688)	<0.001	1.155 (0.865–1.543)	0.328	2.748 (2.138–3.531)	<0.001	1.835 (1.212–2.779)	0.004	7.106 (5.027–10.043)	<0.001	3.741 (2.091–6.693)	<0.001
2012	1.482 (1.246–1.761)	<0.001	1.155 (0.869–1.536)	0.321	3.079 (2.390–3.966)	<0.001	1.981 (1.310–2.995)	0.001	9.127 (6.376–13.066)	<0.001	3.630 (1.959–6.729)	<0.001
2013	1.713 (1.443–2.033)	<0.001	1.463 (1.109–1.931)	0.007	3.538 (2.738–4.573)	<0.001	2.206 (1.447–3.362)	<0.001	15.347 (10.705–22.002)	<0.001	7.012 (3.852–12.767)	<0.001
2014	1.458 (1.214–1.751)	<0.001	0.964 (0.713–1.303)	0.810	3.379 (2.572–4.439)	<0.001	1.997 (1.294–3.082)	0.002	17.095 (11.556–25.289)	<0.001	8.752 (4.681–16.364)	<0.001
2015	1.570 (1.305–1.888)	<0.001	0.848 (0.618–1.163)	0.307	4.326 (3.285–5.695)	<0.001	1.938 (1.226–3.063)	0.005	30.393 (20.183–45.769)	<0.001	9.125 (4.475–18.606)	<0.001
2016	1.566 (1.296–1.892)	<0.001	0.805 (0.585–1.108)	0.184	5.357 (4.048–7.088)	<0.001	2.355 (1.495–3.709)	<0.001	82.814 (53.611–127.926)	<0.001	21.715 (10.294–45.810)	<0.001
2017	1.503 (1.228–1.839)	<0.001	0.841 (0.594–1.190)	0.329	5.908 (4.326–8.068)	<0.001	2.549 (1.514–4.293)	<0.001	613.438 (373.194–1008.338)	<0.001	167.155 (65.640–425.671)	<0.001
2018	1.556 (1.264–1.914)	<0.001	0.794 (0.551–1.145)	0.217	10.077 (7.146–14.211)	<0.001	3.776 (2.116–6.740)	<0.001	1862.299 (864.403–4012.199)	<0.001	647.774 (197.802–2121.368)	<0.001
2019	2.471 (1.993–3.063)	<0.001	1.210 (0.818–1.790)	0.340	63.631 (43.519–93.040)	<0.001	10.244 (4.869–21.551)	<0.001				
2020	4.648 (3.680–5.871)	<0.001	2.104 (1.387–3.192)	<0.001	153.170 (88.140–266.178)	<0.001	69.598 (29.735–162.904)	<0.001				
2021	9.734 (7.487–12.650)	<0.001	5.420 (3.245–9.055)	<0.001								
Stage												
Localized	Ref		Ref		Ref		Ref		Ref		Ref	
Regional	1.933 (1.210–2.116)	<0.001	1.538 (1.346–1.759)	<0.001	1.634 (1.452–1.839)	<0.001	1.504 (1.254–1.803)	<0.001	1.419 (1.223–1.647)	<0.001	1.513 (1.188–1.927)	<0.001
Distant	4.898 (4.392–5.461)	<0.001	3.511 (2.894–4.259)	<0.001	3.549 (3.003–4.194)	<0.001	3.235 (2.404–4.354)	<0.001	2.037 (1.558–2.664)	<0.001	3.101 (1.909–5.037)	<0.001
Unknown	1.696 (1.452–1.981)	<0.001	1.637 (1.199–2.235)	0.002	1.311 (1.059–1.624)	0.013	1.676 (1.081–2.598)	0.021	1.190 (0.908–1.561)	0.207	1.344 (0.697–2.592)	0.377
Primary treatments												
Surgery alone	Ref				Ref				Ref			
Surgery + adjuvant radiotherapy	1.352 (1.210–1.510)	<0.001			1.257 (1.089–1.451)	0.002			1.086 (0.906–1.302)	0.372		
Surgery + adjuvant chemotherapy	1.454 (1.196–1.768)	<0.001			1.283 (0.993–1.657)	0.056			1.192 (0.873–1.626)	0.269		
Radiotherapy alone	3.004 (2.584–3.492)	<0.001			2.569 (2.059–3.204)	<0.001			2.147 (1.581–2.914)	<0.001		
Radiotherapy + adjuvant chemotherapy	2.090 (1.847–2.364)	<0.001			1.748 (1.480–2.066)	<0.001			1.525 (1.236–1.880)	<0.001		
Neoadjuvant chemotherapy + radiotherapy ± adjuvant chemotherapy	2.023 (1.792–2.285)	<0.001			1.757 (1.484–2.080)	<0.001			1.638 (1.315–2.042)	<0.001		
Chemotherapy alone	3.011 (2.649–3.424)	<0.001			2.352 (1.928–2.869)	<0.001			1.747 (1.304–2.340)	<0.001		
Surgery												
Total hysterectomy			Ref				Ref				Ref	
Radical hysterectomy			0.832 (0.637–1.087)	0.177			0.684 (0.452–1.034)	0.071			0.727 (0.322–1.642)	0.443
Trachelectomy (radical or simple)			1.172 (0.684–2.009)	0.563			0.728 (0.295–1.795)	0.490			0.747 (0.148–3.770)	0.724
Conization			0.809 (0.600–1.091)	0.166			0.743 (0.475–1.163)	0.193			0.972 (0.415–2.276)	0.947
Chemotherapy												
Platinum-based chemotherapy			Ref				Ref				Ref	
Other agents			1.161 (0.516–2.610)	0.718			1.004 (0.319–3.160)	0.994			0.622 (0.086–4.506)	0.639
Bevacizumab ± any agents			3.747 (3.030–4.635)	<0.001			3.792 (2.674–5.378)	<0.001			1.059 (0.327–3.424)	0.924
Postoperative VTE												
(−)	Ref		Ref		Ref		Ref		Ref		Ref	
(+)	1.286 (1.097–1.506)	0.002	1.321 (1.002–1.742)	0.049	1.389 (1.104–1.747)	0.005	1.659 (1.144–2.408)	0.008	2.096 (1.560–2.818)	<0.001	2.301 (1.390–3.810)	0.001
Prophylactic anticoagulants												
(−)	Ref		Ref		Ref		Ref		Ref		Ref	
(+)	2.183 (2.033–2.345)	<0.001	2.282 (1.996–2.609)	<0.001	2.291 (2.074–2.530)	<0.001	2.434 (2.021–2.932)	<0.001	2.006 (1.766–2.278)	<0.001	2.195 (1.715–2.809)	<0.001
HRT												
(−)	Ref		Ref		Ref		Ref		Ref		Ref	
(+)	0.655 (0.600–0.715)	<0.001	0.629 (0.549–0.719)	<0.001	0.749 (0.671–0.835)	<0.001	0.753 (0.633–0.896)	0.001	0.734 (0.639–0.844)	<0.001	0.814 (0.643–1.030)	0.086

Abbreviations: CCI, Charlson comorbidity index; CI, confidence interval; HR, hazard ratio; HRT, hormone replacement therapy; SES, socioeconomic status; VTE, venous thromboembolism. Multivariable analyses I and II were adjusted using the same predefined covariate sets as those used throughout the study.

**Table 5:** Sensitivity analysis using stage-stratified Cox regression for OS.

	Localized				Regional				Distant			
	Multivariable Analysis I		Multivariable Analysis II		Multivariable Analysis I		Multivariable Analysis II		Multivariable Analysis I		Multivariable Analysis II	
Variables	HR (95% CI)	*p* Value	HR (95% CI)	*p* Value	HR (95% CI)	*p* Value	HR (95% CI)	*p* Value	HR (95% CI)	*p* Value	HR (95% CI)	*p* Value
Age (years)	0.993 (0.986–1.000)	0.057	0.993 (0.981–1.006)	0.283	0.981 (0.975–0.987)	<0.001	0.982 (0.973–0.991)	<0.001	0.976 (0.968– 0.985)	<0.001	0.981 (0.962–1.000)	0.046
SES												
Low SES	Ref		Ref		Ref		Ref		Ref		Ref	
Mid- or high-SES	0.732 (0.622–0.861)	<0.001	0.785 (0.592–1.040)	0.091	0.840 (0.742–0.951)	0.006	0.833 (0.680–1.022)	0.080	1.082 (0.909–1.287)	0.374	1.038 (0.674–1.598)	0.865
CCI												
0	Ref		Ref		Ref		Ref		Ref		Ref	
1	0.823 (0.676–1.003)	0.054	1.046 (0.763–1.435)	0.780	0.882 (0.760–1.023)	0.100	0.957 (0.766–1.194)	0.696	0.995 (0.799–1.238)	0.962	0.880 (0.555–1.396)	0.589
2	1.072 (0.897–1.282)	0.444	1.011 (0.731–1.397)	0.949	0.797 (0.675–0.942)	0.008	0.735 (0.559–0.968)	0.028	1.038 (0.838–1.286)	0.731	1.610 (0.954–2.718)	0.074
3	0.830 (0.593–1.162)	0.278	0.718 (0.366–1.410)	0.336	1.027 (0.786–1.342)	0.847	0.807 (0.512–1.271)	0.355	0.877 (0.597–1.289)	0.504	0.713 (0.219–2.321)	0.575
≥4	0.989 (0.666–1.468)	0.956	1.155 (0.507–2.633)	0.732	1.264 (0.929–1.721)	0.136	1.581 (0.92–2.717)	0.097	1.361 (0.853–2.172)	0.196	0.603 (0.140–2.590)	0.496
Year of cancer diagnosis												
2007	Ref		Ref		Ref		Ref		Ref		Ref	
2008	2.344 (1.718–3.198)	<0.001	1.736 (1.066–2.826)	0.027	0.962 (0.762–1.215)	0.747	0.679 (0.463–0.995)	0.047	1.083 (0.463–0.995)	0.675	1.595 (0.759–3.352)	0.218
2009	2.147 (1.524–3.024)	<0.001	1.467 (0.842–2.557)	0.176	0.962 (0.752–1.232)	0.761	0.854 (0.589–1.237)	0.403	1.083 (0.463–0.995)	0.679	1.444 (0.628–3.321)	0.387
2010	2.555 (1.810–3.606)	<0.001	1.311 (0.742–2.318)	0.351	0.958 (0.745–1.233)	0.740	0.727 (0.496–1.067)	0.103	1.101 (0.463–0.995)	0.605	1.458 (0.718–2.964)	0.297
2011	2.924 (2.065–4.141)	<0.001	1.595 (0.916–2.777)	0.099	1.070 (0.827–1.384)	0.606	0.911 (0.620–1.340)	0.637	1.103 (0.463–0.995)	0.607	1.239 (0.547–2.810)	0.607
2012	3.212 (2.247–4.591)	<0.001	1.679 (0.974–2.896)	0.062	1.047 (0.812–1.348)	0.724	0.903 (0.618–1.321)	0.600	0.980 (0.463–0.995)	0.913	1.084 (0.471–2.491)	0.850
2013	4.432 (3.122–6.291)	<0.001	2.183 (1.269–3.757)	0.005	1.183 (0.923–1.516)	0.185	1.095 (0.756–1.587)	0.630	1.217 (0.463–0.995)	0.278	1.635 (0.782–3.419)	0.191
2014	3.636 (2.486–5.318)	<0.001	1.534 (0.850–2.766)	0.155	1.018 (0.785–1.320)	0.893	0.696 (0.469–1.035)	0.074	0.941 (0.463–0.995)	0.756	0.812 (0.335–1.967)	0.644
2015	3.607 (2.442–5.329)	<0.001	1.064 (0.563–2.013)	0.848	1.202 (0.923–1.567)	0.172	0.700 (0.457–1.070)	0.100	0.891 (0.463–0.995)	0.541	1.166 (0.539–2.524)	0.697
2016	3.796 (2.543–5.668)	<0.001	1.134 (0.590–2.179)	0.705	1.143 (0.873–1.497)	0.331	0.584 (0.383–0.890)	0.012	0.961 (0.463–0.995)	0.840	0.934 (0.386–2.262)	0.880
2017	3.933 (2.582–5.992)	<0.001	1.249 (0.614–2.539)	0.540	1.055 (0.782–1.422)	0.726	0.642 (0.398–1.034)	0.069	0.846 (0.463–0.995)	0.390	1.269 (0.579–2.778)	0.552
2018	4.677 (2.964–7.380)	<0.001	1.521 (0.768–3.011)	0.229	1.064 (0.781–1.450)	0.694	0.491 (0.299–0.807)	0.005	0.887 (0.463–0.995)	0.529	0.626 (0.224–1.750)	0.372
2019	6.351 (3.873–10.413)	<0.001	1.545 (0.639–3.739)	0.334	1.954 (1.446–2.640)	<0.001	0.884 (0.530–1.474)	0.635	1.024 (0.463–0.995)	0.910	1.506 (0.579–3.916)	0.401
2020	11.168 (6.136–20.327)	<0.001	3.756 (1.457–9.68)	0.006	3.849 (2.756–5.377)	<0.001	1.393 (0.817–2.376)	0.224	1.521 (0.463–0.995)	0.045	1.585 (0.540–4.655)	0.402
2021	58.982 (31.979–108.787)	<0.001	4.445 (1.564–12.631)	0.005	10.354 (6.953–15.418)	<0.001	3.211 (1.548–6.658)	0.002	2.299 (0.463–0.995)	<0.001	7.350 (2.560–21.102)	<0.001
Primary treatments												
Surgery alone	Ref				Ref				Ref			
Surgery + adjuvant radiotherapy	1.639 (1.374–1.956)	<0.001			0.842 (0.716–0.991)	0.039			0.986 (0.739–1.315)	0.925		
Surgery + adjuvant chemotherapy	1.764 (1.309–2.376)	<0.001			0.926 (0.684–1.255)	0.621			0.811 (0.465–1.417)	0.462		
Radiotherapy alone	4.004 (3.013–5.321)	<0.001			1.821 (1.421–2.334)	<0.001			1.670 (1.235–2.259)	<0.001		
Radiotherapy + adjuvant chemotherapy	3.178 (2.544–3.970)	<0.001			1.440 (1.199–1.728)	<0.001			1.107 (0.831–1.475)	0.488		
Neoadjuvant chemotherapy + radiotherapy ± adjuvant chemotherapy	3.528 (2.783–4.473)	<0.001			1.278 (1.075–1.520)	0.005			1.110 (0.837–1.472)	0.469		
Chemotherapy alone	5.135 (4.111–6.414)	<0.001			1.969 (1.599–2.424)	<0.001			2.018 (1.570–2.593)	<0.001		
Surgery												
Total hysterectomy			Ref				Ref				Ref	
Radical hysterectomy			1.023 (0.570–1.834)	0.940			0.701 (0.477–1.029)	0.070			0.729 (0.402–1.322)	0.298
Trachelectomy (radical or simple)			0.828 (0.323–2.123)	0.694			2.071 (0.986–4.349)	0.055			.	.
Conization			0.845 (0.443–1.611)	0.609			0.653 (0.425–1.003)	0.052			1.015 (0.525–1.963)	0.965
Chemotherapy												
Platinum-based chemotherapy			Ref				Ref				Ref	
Other agents			0.977 (0.307–3.107)	0.968			1.300 (0.178–9.500)	0.796			.	.
Bevacizumab ± any agents			6.076 (4.005–9.218)	<0.001			4.596 (3.464–6.099)	<0.001			1.247 (0.774–2.008)	0.364
Postoperative VTE												
(−)	Ref		Ref		Ref		Ref		Ref		Ref	
(+)	2.200 (1.562–3.099)	<0.001	1.488 (0.835–2.65)	0.177	1.431 (1.135–1.804)	0.002	1.431 (0.977–2.094)	0.066	0.763 (0.569–1.024	0.071	1.046 (0.550–1.99)	0.891
Prophylactic anticoagulants												
(−)	Ref		Ref		Ref		Ref		Ref		Ref	
(+)	1.969 (1.730–2.241)	<0.001	2.305 (1.809–2.937)	<0.001	2.436 (2.189–2.711)	<0.001	2.286 (1.906–2.742)	<0.001	1.630 (1.406–1.891)	<0.001	2.116 (1.431–3.129)	<0.001
HRT												
(−)	Ref		Ref		Ref		Ref		Ref		Ref	
(+)	0.614 (0.530–0.712)	<0.001	0.669 (0.529–0.845)	<0.001	0.514 (0.455–0.581)	<0.001	0.532 (0.444–0.637)	<0.001	0.431 (0.353–0.526)	<0.001	0.531 (0.357–0.792)	0.002

Abbreviations: CCI, Charlson comorbidity index; CI, confidence interval; HR, hazard ratio; HRT, hormone replacement therapy; SES, socioeconomic status; VTE, venous thromboembolism. Patients with unknown stage were excluded from the stage-stratified analyses. Multivariable analyses I and II were adjusted using the same predefined covariate sets as those used throughout the study.

## Data Availability

All data generated or analyzed during this study are included in this published article and its information files. The data that support the findings of this study are available from the Health Insurance Review and Assessment Service (HIRA), but restrictions apply to the availability of these data, which were used under license for the current study and so they are not publicly available. The data are available from the authors upon reasonable request and with the permission of the HIRA.

## Supplementary Materials

The following supporting information can be downloaded at: https://www.mdpi.com/article/10.3390/cancers18172841/s1, Figure S1: Graphical assessment of the proportional hazards assumption for HRT use in the 1-, 3-, and 5-year landmark Cox models. Standardized cumulative score process plots are shown for the (A) 1-year, (B) 3-year, and (C) 5-year landmark analyses. The solid gray line represents the observed standardized score process, and the dotted lines represent simulated score processes under the proportional hazards assumption. HRT, hormone replacement therapy; Figure S2: Restricted cubic spline analyses of the association between age and OS. Restricted cubic spline curves show the association between age and the HR for overall survival in (A) the univariable Cox model, (B) multivariable model I, and (C) multivariable model II. The median age (47 years) was used as the reference value (HR = 1.0). Solid black lines represent the estimated hazard ratios, and green dashed lines indicate the 95% confidence intervals. The vertical dotted line indicates the reference age. The *p* values shown in each panel represent the p for non-linearity. HR, hazard ratio; OS, overall survival; Table S1: Conventional Cox proportional hazards models for OS in the original cohort before IPTW; Table S2: Sensitivity analysis of the original cohort after excluding patients with imputed death dates; Table S3: Baseline characteristics at 1-, 3-, and 5-year landmark time points; Table S4: Landmark analyses of the association between HRT duration before each landmark time point and subsequent OS; Table S5: Sensitivity analysis using histology-stratified Cox regression.

## Abbreviations

AC: cervical adenocarcinoma; ASC: adenosquamous carcinoma; CC: cervical cancer; CCI: Charlson Comorbidity Index; CCRT: concurrent chemoradiotherapy; CI: confidence interval; DSS: disease-specific survival; EBRT: external beam radiation therapy; ER: estrogen receptor; ET: estrogen-only therapy; EPT: estrogen–progesterone therapy; FIGO: International Federation of Gynecology and Obstetrics; HIRA: Health Insurance Review and Assessment Service; HR: hazard ratio; HRT: hormone replacement therapy; ICD-10: the 10th revision of the International Statistical Classification of Diseases and Related Health Problems; IPTW: inverse probability of treatment weighting; NC: neuroendocrine carcinoma; NE: not estimable; OS: overall survival; PFS: progression-free survival; PR: progesterone receptor; RFS: recurrence-free survival; SCC: squamous cell carcinoma; SD: standard deviation; SES: socioeconomic status; SMD: standardized mean difference; VB: vaginal brachytherapy; VTE: venous thromboembolism.
