# Supplementary material for: Impact of Post-Primary Treatment Hormone Replacement Therapy on Survival in Women with Cervical Cancer
Source: Cancers (Basel). 2026 Sep 2;18(17):2841. doi: 10.3390/cancers18172841 (PMC13564618; doi:10.3390/cancers18172841)

(A) 1-Year Landmark Analysis

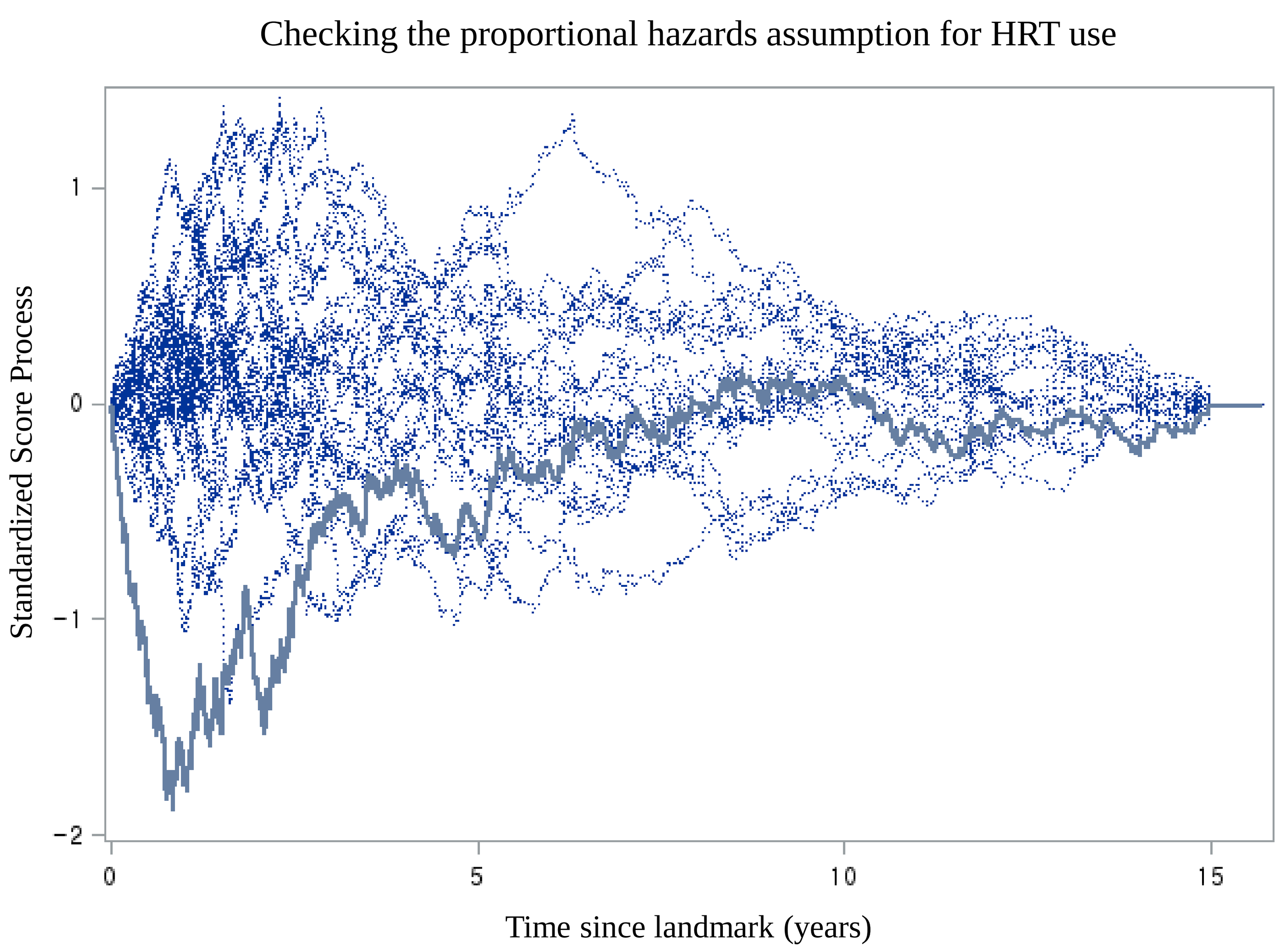

(B) 3-Year Landmark Analysis

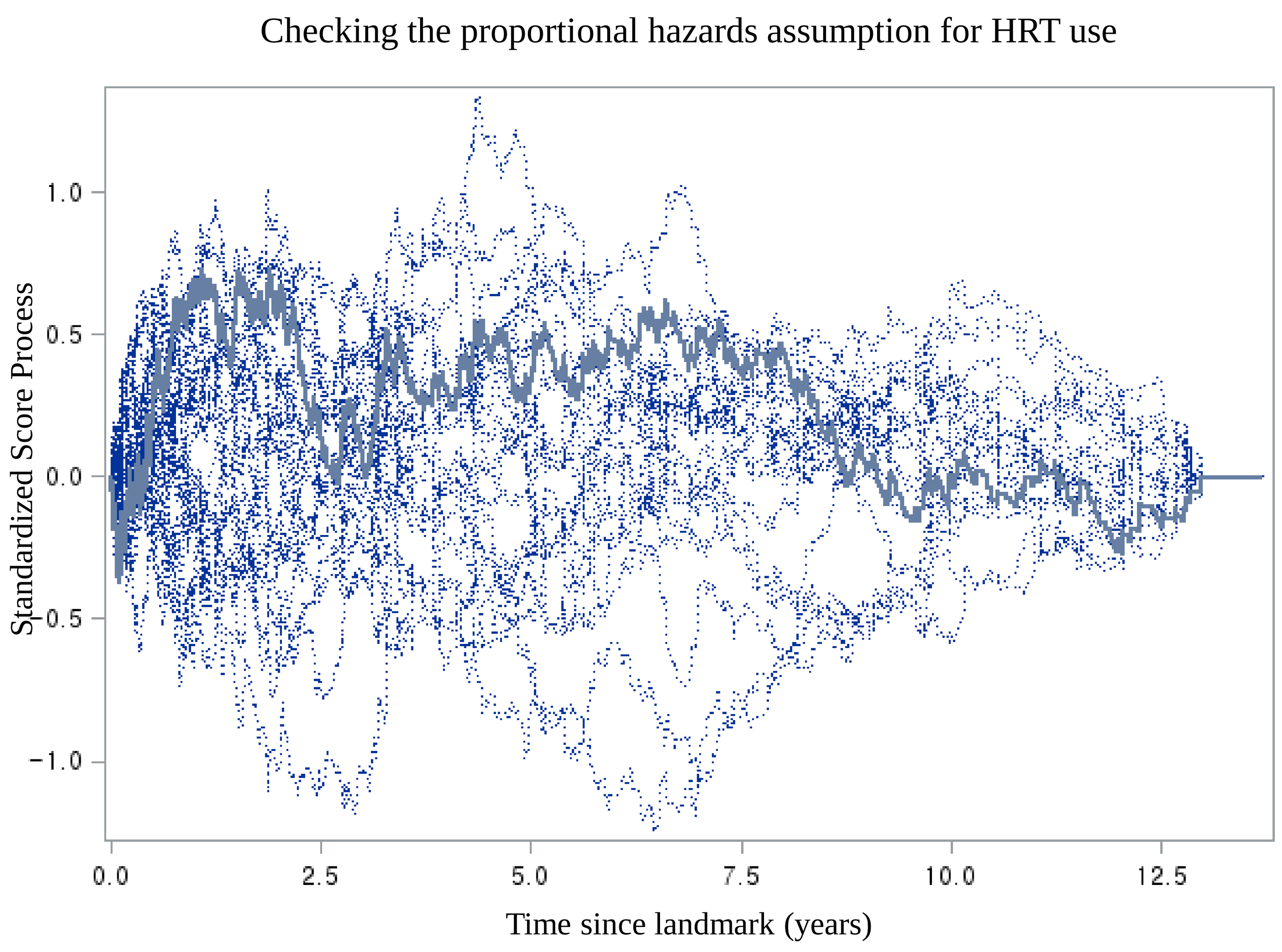

(C) 5-Year Landmark Analysis

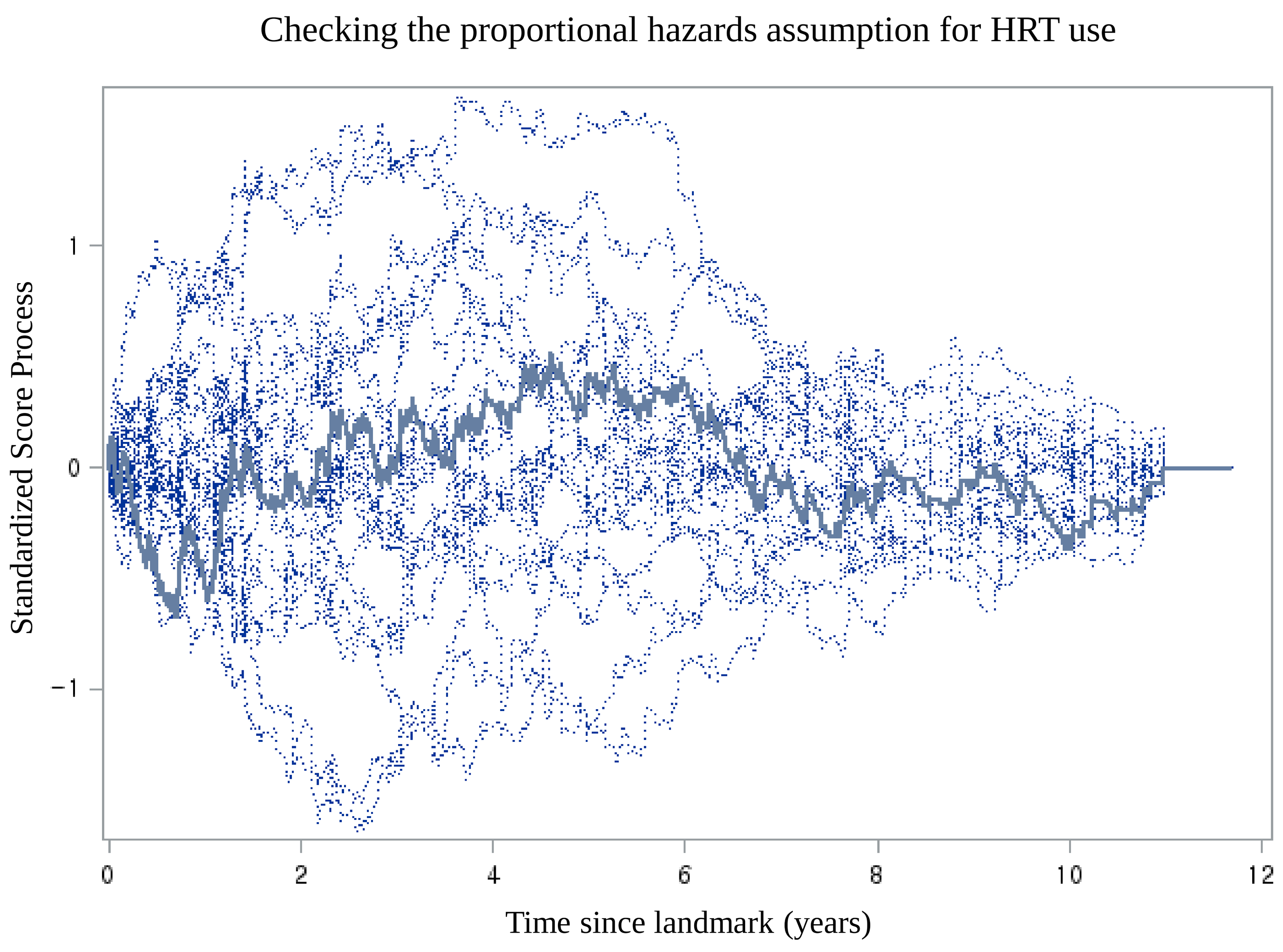

Supplement: Supplementary file 1 [file cancers-18-02841-s001.zip › Figure S1.pdf]
