# Supplementary material for: Impact of Post-Primary Treatment Hormone Replacement Therapy on Survival in Women with Cervical Cancer
Source: Cancers (Basel). 2026 Sep 2;18(17):2841. doi: 10.3390/cancers18172841 (PMC13564618; doi:10.3390/cancers18172841)
Supplement: Supplementary file 1 [file cancers-18-02841-s001.zip › Figure_S2.pdf]

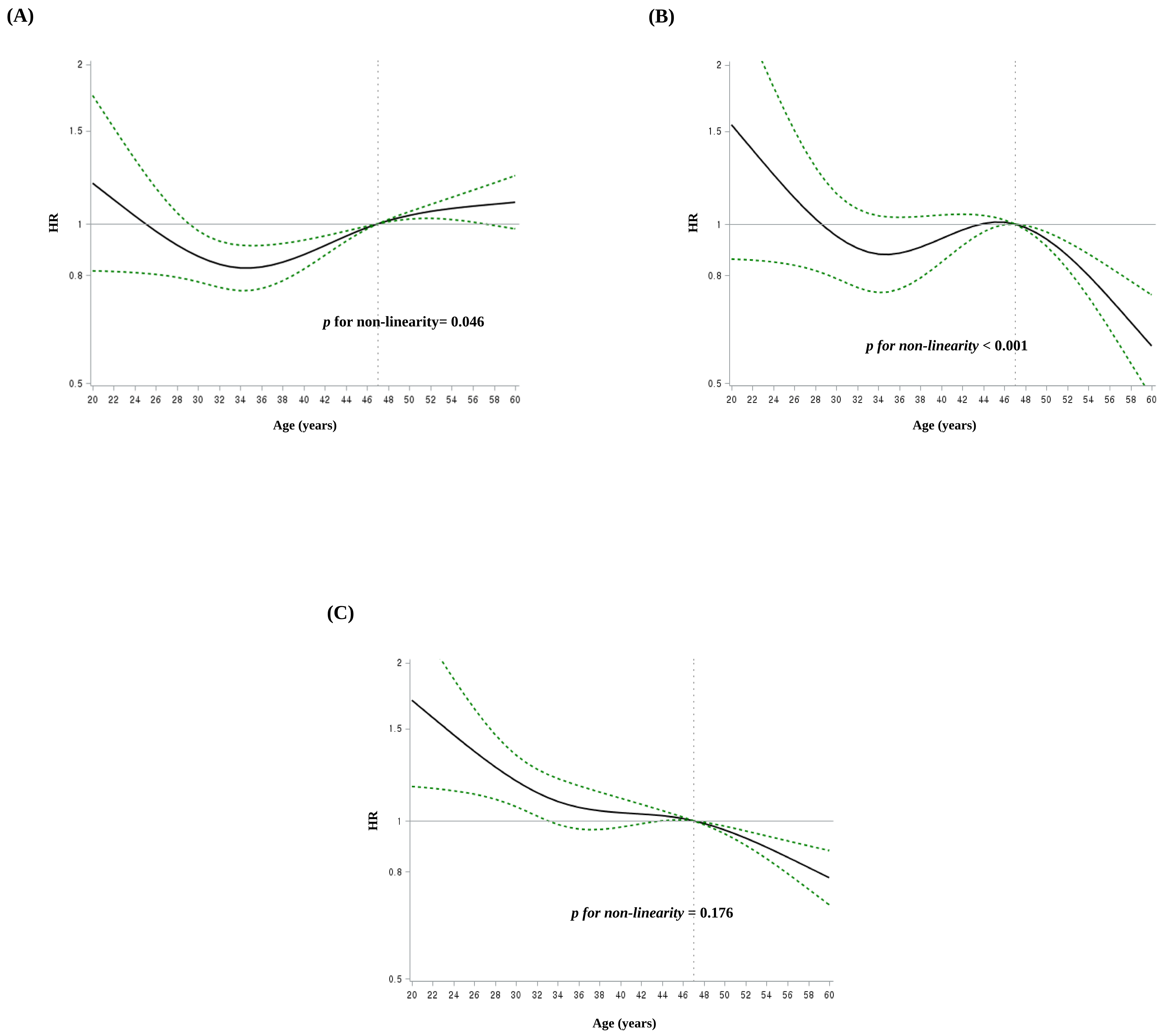
(A)
HR
2
1.5
1
0.8
0.5
20 22 24 26 28 30 32 34 36 38 40 42 44 46 48 50 52 54 56 58 60
Age (years)
p for non-linearity= 0.046
(B)
HR
2
1.5
1
0.8
0.5
20 22 24 26 28 30 32 34 36 38 40 42 44 46 48 50 52 54 56 58 60
Age (years)
p for non-linearity < 0.001
(C)
HR
2
1.5
1
0.8
0.5
20 22 24 26 28 30 32 34 36 38 40 42 44 46 48 50 52 54 56 58 60
Age (years)
p for non-linearity = 0.176
