# Supplementary material for: Impact of Post-Primary Treatment Hormone Replacement Therapy on Survival in Women with Cervical Cancer
Source: Cancers (Basel). 2026 Sep 2;18(17):2841. doi: 10.3390/cancers18172841 (PMC13564618; doi:10.3390/cancers18172841)
Supplement: Supplementary file 1 [file cancers-18-02841-s001.zip › Table S1 (Original Cox).pdf]

Table S1. Conventional Cox proportional hazards models for OS in the original cohort before IPTW.

	Univariable analysis		Multivariable analysis I		Multivariable analysis II	
**Variables**	**HR (95% CI)**	***p* value**	**HR (95% CI)**	***p* value**	**HR (95% CI)**	***p* value**
Age (years)	1.010 (1.006–1.014)	< 0.001	0.984 (0.980–0.988)	< 0.001	0.987 (0.981–0.994)	< 0.001
SES						
Low SES	Ref		Ref		Ref	
Mid- or high-SES	0.678 (0.625–0.736)	< 0.001	0.847 (0.780–0.920)	< 0.001	0.835 (0.720–0.968)	0.017
CCI						
0	Ref		Ref		Ref	
1	0.888 (0.802–0.982)	0.021	0.896 (0.809–0.992)	0.035	0.955 (0.811–1.126)	0.586
2	0.958 (0.867–1.060)	0.406	0.918 (0.83–1.016)	0.099	0.860 (0.714–1.035)	0.111
3	0.960 (0.802–1.149)	0.656	0.884 (0.738–1.058)	0.179	0.823 (0.580–1.167)	0.275
≥4	1.246 (1.015–1.531)	0.036	1.188 (0.965–1.462)	0.104	1.323 (0.878–1.993)	0.182
Year of cancer diagnosis						
2007	Ref		Ref		Ref	
2008	1.229 (1.053–1.434)	0.009	1.204 (1.032–1.404)	0.018	1.014 (0.783–1.315)	0.914
2009	1.315 (1.114–1.551)	0.001	1.177 (0.997–1.388)	0.054	1.033 (0.785–1.359)	0.819
2010	1.339 (1.134–1.582)	< 0.001	1.228 (1.039–1.451)	0.016	0.966 (0.732–1.274)	0.805
2011	1.448 (1.223–1.715)	< 0.001	1.346 (1.136–1.595)	< 0.001	1.099 (0.829–1.457)	0.513
2012	1.601 (1.353–1.894)	< 0.001	1.342 (1.134–1.589)	< 0.001	1.087 (0.823–1.436)	0.555
2013	1.858 (1.576–2.192)	< 0.001	1.596 (1.353–1.884)	< 0.001	1.362 (1.039–1.784)	0.025
2014	1.596 (1.338–1.904)	< 0.001	1.335 (1.117–1.595)	< 0.001	0.886 (0.659–1.191)	0.421
2015	1.779 (1.491–2.122)	< 0.001	1.413 (1.181–1.690)	< 0.001	0.779 (0.572–1.062)	0.114
2016	1.752 (1.462–2.100)	< 0.001	1.401 (1.165–1.685)	< 0.001	0.728 (0.532–0.998)	0.048
2017	1.780 (1.470–2.156)	< 0.001	1.329 (1.093–1.616)	< 0.001	0.793 (0.566–1.111)	0.178
2018	2.048 (1.684–2.491)	< 0.001	1.353 (1.107–1.653)	< 0.001	0.739 (0.517–1.055)	0.096
2019	3.101 (2.534–3.797)	< 0.001	2.160 (1.758–2.653)	< 0.001	1.145 (0.785–1.672)	0.482
2020	6.728 (5.384–8.407)	< 0.001	3.317 (2.646–4.159)	< 0.001	1.724 (1.144–2.596)	0.009
2021	24.270 (19.060–30.910)	< 0.001	6.681 (5.211–8.566)	< 0.001	4.579 (2.82–7.437)	< 0.001
Stage						
Localized	Ref		Ref		Ref	
Regional	2.642 (2.443–2.857)	< 0.001	1.937 (1.772–2.117)	< 0.001	1.557 (1.365–1.778)	< 0.001
Distant	9.862 (9.005–10.800)	< 0.001	4.973 (4.473–5.529)	< 0.001	3.721 (3.084–4.490)	< 0.001
Unknown	1.840 (1.583–2.140)	< 0.001	1.677 (1.439–1.954)	< 0.001	1.629 (1.193–2.224)	0.002
Primary treatments						
Surgery alone	Ref		Ref			
Surgery + adjuvant radiotherapy	1.888 (1.710–2.085)	< 0.001	1.425 (1.278–1.588)	< 0.001		
Surgery + adjuvant chemotherapy	1.702 (1.410–2.054)	< 0.001	1.518 (1.252–1.839)	< 0.001		
Radiotherapy alone	4.637 (4.034–5.330)	< 0.001	3.212 (2.770–3.725)	< 0.001		
Radiotherapy + adjuvant chemotherapy	2.970 (2.669–3.304)	< 0.001	2.281 (2.019–2.576)	< 0.001		
Neoadjuvant chemotherapy + radiotherapy ± adjuvant chemotherapy	3.108 (2.794–3.458)	< 0.001	2.178 (1.931–2.457)	< 0.001		
Chemotherapy alone	7.587 (6.815–8.445)	< 0.001	3.442 (3.038–3.899)	< 0.001		
Surgery						
Total hysterectomy	Ref				Ref	
Radical hysterectomy	0.632 (0.506–0.789)	< 0.001			0.837 (0.642–1.090)	0.186
Trachelectomy (radical or simple)	0.727 (0.471–1.121)	0.149			1.219 (0.713–2.084)	0.470
Conization	0.415 (0.327–0.527)	< 0.001			0.820 (0.610–1.102)	0.187
Radiotherapy						
EBRT ± VB	Ref					
VB alone	1.089 (0.991–1.196)	0.078				
CCRT	1.044 (0.835–1.306)	0.707				
Chemotherapy						
Platinum-based chemotherapy	Ref				Ref	

Other agents	1.148 (0.635–2.075)	0.649			1.095 (0.487–2.461)	0.826
Bevacizumab ± any agents	4.495 (4.096–4.933)	< 0.001			3.551 (2.883–4.372)	< 0.001
Postoperative VTE						
(-)	Ref		Ref		Ref	
(+)	2.945 (2.532–3.424)	< 0.001	1.190 (1.018–1.391)	0.029	1.266 (0.962–1.665)	0.092
Prophylactic anticoagulants						
(-)	Ref		Ref		Ref	
(+)	2.321 (2.172–2.480)	< 0.001	2.138 (1.995–2.291)	< 0.001	2.297 (2.014–2.621)	< 0.001
HRT						
(-)	Ref		Ref		Ref	
(+)	0.583 (0.538–0.631)	< 0.001	0.525 (0.484–0.570)	< 0.001	0.578 (0.507–0.658)	< 0.001

Abbreviations: CCI, Charlson comorbidity index; CCRT, concurrent chemoradiotherapy; CI, confidence interval; EBRT, external beam radiation therapy; HR, hazard ratio; HRT, hormone replacement therapy; OS, overall survival; SES, socioeconomic status; VB, vaginal brachytherapy; VTE, venous thromboembolism.
Multivariable analyses I and II were adjusted using the same predefined covariate sets as those used throughout the study.
