# Supplementary material for: Impact of Post-Primary Treatment Hormone Replacement Therapy on Survival in Women with Cervical Cancer
Source: Cancers (Basel). 2026 Sep 2;18(17):2841. doi: 10.3390/cancers18172841 (PMC13564618; doi:10.3390/cancers18172841)
Supplement: Supplementary file 1 [file cancers-18-02841-s001.zip › Table S2 (HIRA_death_Cox).pdf]

Table S2. Sensitivity analysis of the original cohort after excluding patients with imputed death dates.

Variables	Univariable analysis		Multivariable analysis I		Multivariable analysis II	
	HR (95% CI)	*p* value	HR (95% CI)	*p* value	HR (95% CI)	*p* value
Age (years)	1.016 (1.011–1.020)	< 0.001	0.990 (0.985–0.995)	< 0.001	0.988 (0.980–0.995)	0.001
SES						
Low SES	Ref		Ref		Ref	
Mid- or high-SES	0.664 (0.603–0.730)	< 0.001	0.887 (0.805–0.977)	0.015	0.829 (0.701–0.980)	0.028
CCI						
0	Ref		Ref		Ref	
1	0.942 (0.838–1.059)	0.319	0.973 (0.864–1.095)	0.646	1.007 (0.837–1.211)	0.942
2	0.986 (0.875–1.112)	0.823	0.926 (0.821–1.045)	0.214	0.917 (0.743–1.133)	0.422
3	0.975 (0.788–1.206)	0.813	0.867 (0.700–1.074)	0.192	0.902 (0.613–1.329)	0.603
≥4	1.322 (1.045–1.674)	0.020	1.196 (0.943–1.517)	0.141	1.553 (1.000–2.412)	0.050
Year of cancer diagnosis						
2007	Ref		Ref		Ref	
2008	1.217 (1.011–1.465)	0.038	1.208 (1.003–1.454)	0.046	1.206 (0.885–1.643)	0.235
2009	1.142 (0.938–1.392)	0.186	1.009 (0.828–1.230)	0.929	1.028 (0.741–1.426)	0.868
2010	1.146 (0.942–1.394)	0.174	1.047 (0.860–1.276)	0.646	1.078 (0.781–1.489)	0.646
2011	1.201 (0.986–1.463)	0.069	1.089 (0.893–1.328)	0.400	1.200 (0.868–1.660)	0.269
2012	1.195 (0.980–1.457)	0.079	1.010 (0.827–1.234)	0.919	1.073 (0.775–1.484)	0.672
2013	1.417 (1.170–1.716)	< 0.001	1.200 (0.989–1.456)	0.065	1.415 (1.037–1.930)	0.029
2014	1.187 (0.968–1.457)	0.100	0.976 (0.793–1.201)	0.818	0.802 (0.567–1.134)	0.212
2015	1.296 (1.057–1.589)	0.013	1.024 (0.832–1.261)	0.821	0.654 (0.456–0.939)	0.021
2016	1.178 (0.954–1.456)	0.128	0.936 (0.754–1.162)	0.549	0.599 (0.415–0.865)	0.006
2017	1.208 (0.968–1.507)	0.095	0.900 (0.718–1.129)	0.363	0.664 (0.453–0.974)	0.036
2018	1.413 (1.132–1.763)	0.002	0.923 (0.735–1.160)	0.494	0.580 (0.387–0.870)	0.008
2019	1.846 (1.470–2.318)	< 0.001	1.341 (1.063–1.694)	0.014	0.789 (0.515–1.206)	0.274
2020	3.027 (2.369–3.868)	< 0.001	1.779 (1.384–2.286)	< 0.001	1.219 (0.788–1.886)	0.374
2021	10.670 (8.175–13.93)	< 0.001	3.804 (2.904–4.983)	< 0.001	2.982 (1.782–4.989)	< 0.001
Stage						
Localized	Ref		Ref		Ref	
Regional	3.185 (2.885–3.516)	< 0.001	2.162 (1.936–2.414)	< 0.001	1.555 (1.333–1.815)	< 0.001
Distant	11.700 (10.490–13.050)	< 0.001	5.459 (4.814–6.191)	< 0.001	3.905 (3.174–4.806)	< 0.001
Unknown	2.176 (1.803–2.627)	< 0.001	1.854 (1.532–2.245)	< 0.001	1.818 (1.277–2.588)	< 0.001
Primary treatments						
Surgery alone	Ref		Ref			
Surgery + adjuvant radiotherapy	2.474 (2.184–2.803)	< 0.001	1.644 (1.435–1.882)	< 0.001		
Surgery + adjuvant chemotherapy	2.168 (1.716–2.738)	< 0.001	1.655 (1.304–2.101)	< 0.001		
Radiotherapy alone	6.084 (5.153–7.183)	< 0.001	3.532 (2.960–4.215)	< 0.001		
Radiotherapy + adjuvant chemotherapy	3.800 (3.324–4.346)	< 0.001	2.521 (2.169–2.931)	< 0.001		
Neoadjuvant chemotherapy + radiotherapy ± adjuvant chemotherapy	3.820 (3.348–4.359)	< 0.001	2.517 (2.173–2.916)	< 0.001		
Chemotherapy alone	8.967 (7.872–10.210)	< 0.001	3.695 (3.180–4.293)	< 0.001		
Surgery						
Total hysterectomy	Ref				Ref	
Radical hysterectomy	0.803 (0.609–1.057)	0.118			0.959 (0.713–1.289)	0.781
Trachelectomy (radical or simple)	0.633 (0.352–1.140)	0.128			1.235 (0.675–2.261)	0.494
Conization	0.364 (0.268–0.494)	< 0.001			0.853 (0.610–1.192)	0.352
Radiotherapy						
EBRT ± VB	Ref					
VB alone	1.018 (0.913–1.135)	0.745				
CCRT	1.076 (0.829–1.395)	0.582				
Chemotherapy						
Platinum-based chemotherapy	Ref				Ref	

Other agents	1.081 (0.540–2.165)	0.825			1.241 (0.510–3.017)	0.634
Bevacizumab ± any agents	4.458 (4.032–4.930)	< 0.001			4.309 (3.437–5.402)	< 0.001
Postoperative VTE						
(-)	Ref		Ref		Ref	
(+)	3.170 (2.676–3.755)	< 0.001	1.146 (0.964–1.363)	0.122	1.210 (0.888–1.647)	0.227
Prophylactic anticoagulants						
(-)	Ref		Ref		Ref	
(+)	3.048 (2.806–3.312)	< 0.001	2.814 (2.582–3.066)	< 0.001	2.796 (2.383–3.279)	< 0.001
HRT						
(-)	Ref		Ref		Ref	
(+)	0.614 (0.559–0.675)	< 0.001	0.578 (0.524–0.637)	< 0.001	0.551 (0.473–0.641)	< 0.001

Abbreviations: CCI, Charlson comorbidity index; CCRT, concurrent chemoradiotherapy; CI, confidence interval; EBRT, external beam radiation therapy; HR, hazard ratio; HRT, hormone replacement therapy; SES, socioeconomic status; VB, vaginal brachytherapy; VTE, venous thromboembolism.

Multivariable analyses I and II were adjusted using the same predefined covariate sets as those used throughout the study.
