# Supplementary material for: Impact of Post-Primary Treatment Hormone Replacement Therapy on Survival in Women with Cervical Cancer
Source: Cancers (Basel). 2026 Sep 2;18(17):2841. doi: 10.3390/cancers18172841 (PMC13564618; doi:10.3390/cancers18172841)
Supplement: Supplementary file 1 [file cancers-18-02841-s001.zip › Table S3 (Landmark baseline).pdf]

Table S3. Baseline characteristics at 1-, 3-, and 5-year landmark time points.

	1 year				3 year				5 year			
	No. (%)				No. (%)				No. (%)			
Characteristics	Total	HRT (-)	HRT (+)	*p* value	Total	HRT (-)	HRT (+)	*p* value	Total	HRT (-)	HRT (+)	*p* value
No. of women	14,343 (100)	11,091 (77.3)	3,252 (22.7)		11,246 (100)	8,076 (71.8)	3,170 (28.2)		8,998 (100)	6,263 (69.6)	2,735 (30.4)	
Age (years)	46.2 ± 8.7	46.4 ± 9.0	45.5 ± 7.4	< 0.001	46.1 ± 8.6	46.3 ± 9.0	45.5 ± 7.4	< 0.001	46.0 ± 8.6	46.1 ± 9.1	45.7 ± 7.4	0.010
SES												
Low SES	2,075 (14.5)	1,569 (14.1)	506 (15.6)	0.044	1,546 (13.7)	1,051 (13.0)	495 (15.6)	< 0.001	1,212 (13.5)	783 (12.5)	429 (15.7)	< 0.001
Mid- or high-SES	12,268 (85.5)	9,522 (85.9)	2,746 (84.4)	0.044	9,700 (86.3)	7,025 (87.0)	2,675 (84.4)	< 0.001	7,786 (86.5)	5,480 (87.5)	2,306 (84.3)	< 0.001
CCI												
0	9,851 (68.7)	7,608 (68.6)	2,243 (69.0)		7,729 (68.7)	5,535 (68.5)	2,194 (69.2)		6,241 (69.4)	4,329 (69.1)	1,912 (69.9)	
1	1,969 (13.7)	1,533 (13.8)	436 (13.4)		1,543 (13.7)	1,128 (14.0)	415 (13.1)		1,183 (13.1)	829 (13.2)	354 (12.9)	
2	1,720 (12.0)	1,325 (11.9)	395 (12.1)	0.958	1,345 (12.0)	956 (11.8)	389 (12.3)	0.730	1,086 (12.1)	762 (12.2)	324 (11.8)	0.932
3	498 (3.5)	386 (3.5)	112 (3.4)		399 (3.5)	289 (3.6)	110 (3.5)		311 (3.5)	216 (3.4)	95 (3.5)	
≥4	305 (2.1)	239 (2.2)	66 (2.0)		230 (2.0)	168 (2.1)	62 (2.0)		177 (2.0)	127 (2.0)	50 (1.8)	
Year of cancer diagnosis												
2007	1,105 (7.7)	768 (6.9)	337 (10.4)	< 0.001	958 (8.5)	602 (7.5)	356 (11.2)	< 0.001	900 (10.0)	561 (9.0)	339 (12.4)	< 0.001
2008	1,192 (8.3)	873 (7.9)	319 (9.8)	< 0.001	1,048 (9.3)	708 (8.8)	340 (10.7)	0.001	981 (10.9)	650 (10.4)	331 (12.1)	0.016
2009	1,015 (7.1)	746 (6.7)	269 (8.3)	0.003	885 (7.9)	601 (7.4)	284 (9.0)	0.007	835 (9.3)	563 (9.0)	272 (9.9)	0.151
2010	1,087 (7.6)	806 (7.3)	281 (8.6)	0.009	976 (8.7)	676 (8.4)	300 (9.5)	0.064	921 (10.2)	628 (10.0)	293 (10.7)	0.324
2011	1,054 (7.3)	802 (7.2)	252 (7.7)	0.320	945 (8.4)	675 (8.4)	270 (8.5)	0.784	891 (9.9)	626 (10.0)	265 (9.7)	0.655
2012	1,087 (7.6)	824 (7.4)	263 (8.1)	0.213	961 (8.5)	675 (8.4)	286 (9.0)	0.257	897 (10.0)	619 (9.9)	278 (10.2)	0.682
2013	1,121 (7.8)	860 (7.8)	261 (8.0)	0.612	971 (8.6)	699 (8.7)	272 (8.6)	0.899	926 (10.3)	664 (10.6)	262 (9.6)	0.142
2014	1,042 (7.3)	786 (7.1)	256 (7.9)	0.129	927 (8.2)	667 (8.3)	260 (8.2)	0.921	880 (9.8)	625 (10.0)	255 (9.3)	0.336
2015	1,022 (7.1)	812 (7.3)	210 (6.5)	0.092	909 (8.1)	683 (8.5)	226 (7.1)	0.020	852 (9.5)	633 (10.1)	219 (8.0)	0.002
2016	1,032 (7.2)	839 (7.6)	193 (5.9)	0.002	933 (8.3)	719 (8.9)	214 (6.8)	< 0.001	870 (9.7)	655 (10.5)	215 (7.9)	< 0.001
2017	939 (6.5)	769 (6.9)	170 (5.2)	< 0.001	844 (7.5)	659 (8.2)	185 (5.8)	< 0.001	36 (0.4)	31 (0.5)	5 (0.2)	0.031
2018	931 (6.5)	781 (7.0)	150 (4.6)	< 0.001	826 (7.3)	658 (8.1)	168 (5.3)	< 0.001	9 (0.1)	8 (0.1)	1 (0.0)	0.208 [a]
2019	839 (5.8)	680 (6.1)	159 (4.9)	0.008	45 (0.4)	40 (0.5)	5 (0.2)	0.011				
2020	787 (5.5)	660 (6.0)	127 (3.9)	< 0.001	18 (0.2)	14 (0.2)	4 (0.1)	0.573 [a]				

2021	90 (0.6)	85 (0.8)	5 (0.2)	<0.001								
Histologic types												
SCC	12,201 (85.1)	9,458 (85.3)	2,743 (84.3)		9,614 (85.5)	6,918 (85.7)	2,696 (85.0)		7,686 (85.4)	5,362 (85.6)	2,324 (85.0)	
AC	1,436 (10.0)	1,104 (10.0)	332 (10.2)		1,112 (9.9)	797 (9.9)	315 (9.9)		906 (10.1)	627 (10.0)	279 (10.2)	
ASC	564 (3.9)	411 (3.7)	153 (4.7)	0.039	452 (4.0)	317 (3.9)	135 (4.3)	0.408	366 (4.1)	247 (3.9)	119 (4.4)	0.798
Lage cell NC	33 (0.2)	29 (0.3)	4 (0.1)		17 (0.2)	9 (0.1)	8 (0.3)		15 (0.2)	9 (0.1)	6 (0.2)	
Small cell NC	109 (0.8)	89 (0.8)	20 (0.6)		51 (0.5)	35 (0.4)	16 (0.5)		25 (0.3)	18 (0.3)	7 (0.3)	
Stage												
Localized	7,385 (51.5)	5,725 (51.6)	1,660 (51.0)	0.565	6,325 (56.2)	4,653 (57.6)	1,672 (52.7)	< 0.001	5,258 (58.4)	3,772 (60.2)	1,486 (54.3)	< 0.001
Regional	5,025 (35.0)	3,755 (33.9)	1,270 (39.1)	< 0.001	3,792 (33.7)	2,587 (32.0)	1,205 (38.0)	< 0.001	2,945 (32.7)	1,922 (30.7)	1,023 (37.4)	< 0.001
Distant	1,176 (8.2)	988 (8.9)	188 (5.8)	< 0.001	532 (4.7)	374 (4.6)	158 (5.0)	0.427	299 (3.3)	194 (3.1)	105 (3.8)	0.071
Unknown	757 (5.3)	623 (5.6)	134 (4.1)	< 0.001	597 (5.3)	462 (5.7)	135 (4.3)	0.002	496 (5.5)	375 (6.0)	121 (4.4)	0.003
Primary treatments												
Surgery alone	5,929 (41.3)	4,926 (44.4)	1,003 (30.8)	< 0.001	5,025 (44.7)	4,054 (50.2)	971 (30.6)	< 0.001	4,105 (45.6)	3,262 (52.1)	843 (30.8)	< 0.001
Surgery + adjuvant radiotherapy	3,126 (21.8)	2,025 (18.3)	1,101 (33.9)	< 0.001	2,542 (22.6)	1,550 (19.2)	992 (31.3)	< 0.001	2,070 (23.0)	1,215 (19.4)	855 (31.3)	< 0.001
Surgery + adjuvant chemotherapy	474 (3.3)	324 (2.9)	150 (4.6)	< 0.001	410 (3.6)	261 (3.2)	149 (4.7)	< 0.001	373 (4.1)	238 (3.8)	135 (4.9)	< 0.001
Radiotherapy alone	506 (3.5)	429 (3.9)	77 (2.4)	< 0.001	337 (3.0)	236 (2.9)	101 (3.2)	< 0.001	265 (2.9)	176 (2.8)	89 (3.3)	< 0.001
Radiotherapy + adjuvant chemotherapy	1,418 (9.9)	1,051 (9.5)	367 (11.3)	< 0.001	1,130 (10.0)	719 (8.9)	411 (13.0)	< 0.001	1,003 (11.1)	609 (9.7)	394 (14.4)	< 0.001
Neoadjuvant chemotherapy + radiotherapy ± adjuvant chemotherapy	1,864 (13.0)	1,458 (13.1)	406 (12.5)	< 0.001	1,275 (11.3)	886 (11.0)	389 (12.3)	< 0.001	871 (9.7)	564 (9.0)	307 (11.2)	< 0.001
Chemotherapy alone	1,026 (7.2)	878 (7.9)	148 (4.6)	<0.001	527 (4.7)	370 (4.6)	157 (5.0)	< 0.001	311 (3.5)	199 (3.2)	112 (4.1)	< 0.001
Surgery												
Total hysterectomy	417 (2.9)	332 (3.0)	85 (2.6)	0.257	267 (2.4)	202 (2.5)	65 (2.1)	0.158	130 (1.4)	92 (1.5)	38 (1.4)	0.771
Radical hysterectomy	6,207 (43.3)	4,450 (40.1)	1,757 (54.0)	< 0.001	5,218 (46.4)	3,582 (44.4)	1,636 (51.6)	< 0.001	4,397 (48.9)	2,964 (47.3)	1,433 (52.4)	< 0.001
Trachelectomy (radical or simple)	143 (1.0)	126 (1.1)	17 (0.5)	0.002	115 (1.0)	98 (1.2)	17 (0.5)	0.001	87 (1.0)	71 (1.1)	16 (0.6)	0.014
Conization	2,762 (19.3)	2,367 (21.3)	395 (12.1)	< 0.001	2,377 (21.1)	1,983 (24.6)	394 (12.4)	< 0.001	1,934 (21.5)	1,588 (25.4)	346 (12.7)	< 0.001
Radiotherapy												
EBRT ± VB	2,029 (14.1)	1,563 (14.1)	466 (14.3)	0.733	1,560 (13.9)	1,029 (12.7)	531 (16.8)	< 0.001	1,358 (15.1)	856 (13.7)	502 (18.4)	< 0.001
VB alone	1,600 (11.2)	1,269 (11.4)	331 (10.2)	0.044	1,046 (9.3)	734 (9.1)	312 (9.8)	0.216	658 (7.3)	427 (6.8)	231 (8.4)	0.006
CCRT	159 (1.1)	106 (1.0)	53 (1.6)	0.001	136 (1.2)	78 (1.0)	58 (1.8)	< 0.001	123 (1.4)	66 (1.1)	57 (2.1)	< 0.001
Chemotherapy												
Platinum-based chemotherapy	782 (5.5)	649 (5.9)	133 (4.1)	< 0.001	445 (4.0)	298 (3.7)	147 (4.6)	0.020	283 (3.1)	177 (2.8)	106 (3.9)	0.009

Other agents	3 (0.0)	3 (0.0)	0 (0.0)	0.348 [a]	2 (0.0)	2 (0.0)	0 (0.0)	0.376 [a]	1 (0.0)	1 (0.0)	0 (0.0)	0.509 [a]
Bevacizumab ± any agents	241 (1.7)	226 (2.0)	15 (0.5)	<0.001	80 (0.7)	70 (0.9)	10 (0.3)	0.002	27 (0.3)	21 (0.3)	6 (0.2)	0.355
Postoperative VTE												
(-)	14,056 (98.0)	10,856 (97.9)	3,200 (98.4)	0.063	11,063 (98.4)	7,935 (98.3)	3,128 (98.7)	0.112	8,872 (98.6)	6,174 (98.6)	2,698 (98.6)	0.800
(+)	287 (2.0)	235 (2.1)	52 (1.6)	0.063	183 (1.6)	141 (1.7)	42 (1.3)	0.112	126 (1.4)	89 (1.4)	37 (1.4)	0.800
Prophylactic anticoagulants												
(-)	7,954 (55.5)	6,264 (56.5)	1,690 (52.0)	<0.001	6,477 (57.6)	4,756 (58.9)	1,721 (54.3)	< 0.001	5,374 (59.7)	3,817 (60.9)	1,557 (56.9)	< 0.001
(+)	6,389 (44.5)	4,827 (43.5)	1,562 (48.0)	<0.001	4,769 (42.4)	3,320 (41.1)	1,449 (45.7)	< 0.001	3,624 (40.3)	2,446 (39.1)	1,178 (43.1)	< 0.001
**HRT-related characteristics among HRT users only [b]**												
	**1 year**				**3 year**				**5 year**			
**Characteristics**			**HRT (+)**				**HRT (+)**				**HRT (+)**	
HRT formulation [c]												
ET			1,680 (51.7)				1,618 (51.0)				1,380 (50.5)	
EPT			2,209 (67.9)				2,167 (68.4)				1,873 (68.5)	
Tibolone			1,043 (32.1)				1,003 (31.6)				862 (31.5)	
HRT duration up to the landmark time point (years)												
≤0.5			2,225 (68.4)				1,911 (60.3)				1,610 (58.9)	
0.5–1			1,027 (31.6)				941 (29.7)				783 (28.6)	
1–2							215 (6.8)				169 (6.2)	
2–3							103 (3.2)				100 (3.7)	
3–4											40 (1.5)	
4–5											33 (1.2)	
Time from primary treatment completion to HRT initiation (years), mean (SD)			0.3 ± 0.3				0.5 ± 0.5				0.6 ± 0.8	

Abbreviations: AC, cervical adenocarcinoma; ASC, adenosquamous carcinoma; CCI, Charlson comorbidity index; CCRT, concurrent chemoradiotherapy; EBRT, external beam radiation therapy; ET, estrogen-only therapy; EPT, estrogen-progesterone therapy; HRT, hormone replacement therapy; NC, neuroendocrine carcinoma; No, number; SES, socioeconomic status; SCC, squamous cell carcinoma; SD, standard deviation; VB, vaginal brachytherapy; VTE, venous thromboembolism.

[a] Fisher's exact test was used for this analysis.

[b] HRT-related characteristics are presented descriptively only for women who received HRT and were not included in the baseline balance comparison between the HRT and non-HRT groups. HRT-related variables were applicable only to the HRT group and are therefore presented in the HRT (+) column.

[c] The HRT formulation categories were not mutually exclusive because some women received more than one HRT formulation during follow-up.
