# Supplementary material for: Impact of Post-Primary Treatment Hormone Replacement Therapy on Survival in Women with Cervical Cancer
Source: Cancers (Basel). 2026 Sep 2;18(17):2841. doi: 10.3390/cancers18172841 (PMC13564618; doi:10.3390/cancers18172841)
Supplement: Supplementary file 1 [file cancers-18-02841-s001.zip › Table S4 (Landmark Cox_HRT_ Duration).pdf]

Table S4. Landmark analyses of the association between HRT duration before each landmark time point and subsequent OS.

	1 year				3 year				5 year			
	Multivariable analysis III		Multivariable analysis IV		Multivariable analysis III		Multivariable analysis IV		Multivariable analysis III		Multivariable analysis IV	
Variables	HR (95% CI)	*p* value	HR (95% CI)	*p* value	HR (95% CI)	*p* value	HR (95% CI)	*p* value	HR (95% CI)	*p* value	HR (95% CI)	*p* value
Age (years)	0.987 (0.983–0.991)	< 0.001	0.988 (0.981–0.994)	< 0.001	0.994 (0.989–1.000)	0.051	0.999 (0.990–1.009)	0.918	0.998 (0.991–1.005)	0.612	1.006 (0.992–1.020)	0.399
SES												
Low SES	Ref		Ref		Ref		Ref		Ref		Ref	
Mid- or high-SES	0.875 (0.803–0.952)	0.002	0.855 (0.736–0.994)	0.042	0.881 (0.779–0.996)	0.043	0.846 (0.686–1.043)	0.117	0.909 (0.770–1.072)	0.256	0.759 (0.572–1.008)	0.057
CCI												
0	Ref		Ref		Ref		Ref		Ref		Ref	
1	0.909 (0.819–1.008)	0.072	0.971 (0.823–1.147)	0.733	0.899 (0.777–1.039)	0.148	1.050 (0.836–1.318)	0.677	0.768 (0.628–0.938)	0.010	0.891 (0.636–1.25)	0.505
2	0.935 (0.843–1.037)	0.204	0.874 (0.724–1.056)	0.163	0.929 (0.802–1.075)	0.321	0.901 (0.69–1.177)	0.446	0.875 (0.725–1.057)	0.167	0.840 (0.580–1.216)	0.356
3	0.899 (0.747–1.080)	0.255	0.860 (0.606–1.220)	0.398	1.016 (0.793–1.301)	0.900	0.814 (0.506–1.309)	0.396	0.995 (0.717–1.380)	0.974	0.923 (0.487–1.747)	0.805
≥4	1.184 (0.953–1.470)	0.128	1.340 (0.881–2.037)	0.171	1.087 (0.791–1.493)	0.608	1.635 (0.937–2.855)	0.084	1.298 (0.891–1.891)	0.174	1.869 (0.956–3.655)	0.068
Year of cancer diagnosis												
2007	Ref		Ref		Ref		Ref		Ref		Ref	
2008	1.218 (1.039–1.427)	0.015	1.058 (0.811–1.381)	0.678	1.608 (1.290–2.005)	< 0.001	1.287 (0.884–1.875)	0.188	2.250 (1.688–2.999)	< 0.001	1.419 (0.854–2.355)	0.176
2009	1.234 (1.041–1.463)	0.015	1.106 (0.836–1.463)	0.482	1.753 (1.372–2.240)	< 0.001	1.577 (1.058–2.350)	0.025	3.022 (2.181–4.188)	< 0.001	2.403 (1.397–4.133)	0.002
2010	1.302 (1.096–1.546)	0.003	1.022 (0.769–1.360)	0.879	2.317 (1.814–2.960)	< 0.001	1.682 (1.125–2.513)	0.011	4.950 (3.547–6.908)	< 0.001	2.786 (1.587–4.889)	< 0.001
2011	1.419 (1.192–1.689)	< 0.001	1.158 (0.867–1.546)	0.322	2.751 (2.141–3.535)	< 0.001	1.844 (1.218–2.793)	0.004	7.121 (5.033–10.076)	< 0.001	3.778 (2.111–6.761)	< 0.001
2012	1.482 (1.246–1.762)	< 0.001	1.160 (0.872–1.543)	0.308	3.089 (2.398–3.979)	< 0.001	2.018 (1.334–3.053)	0.001	9.150 (6.385–13.110)	< 0.001	3.706 (1.999–6.873)	< 0.001
2013	1.715 (1.445–2.036)	< 0.001	1.468 (1.113–1.938)	0.007	3.555 (2.750–4.595)	< 0.001	2.208 (1.448–3.366)	< 0.001	15.381 (10.720–22.069)	< 0.001	7.060 (3.877–12.855)	< 0.001
2014	1.460 (1.215–1.753)	< 0.001	0.969 (0.717–1.311)	0.840	3.401 (2.589–4.469)	< 0.001	2.037 (1.319–3.146)	0.001	17.133 (11.571–25.369)	< 0.001	8.952 (4.782–16.759)	< 0.001
2015	1.569 (1.305–1.887)	< 0.001	0.850 (0.620–1.167)	0.315	4.340 (3.296–5.715)	< 0.001	1.956 (1.238–3.093)	0.004	30.460 (20.210–45.908)	< 0.001	9.259 (4.539–18.887)	< 0.001
2016	1.567 (1.297–1.893)	< 0.001	0.809 (0.587–1.113)	0.193	5.368 (4.057–7.103)	< 0.001	2.393 (1.519–3.768)	< 0.001	82.823 (53.570–128.048)	< 0.001	21.990 (10.424–46.387)	< 0.001
2017	1.502 (1.228–1.838)	< 0.001	0.844 (0.596–1.194)	0.338	5.923 (4.336–8.089)	< 0.001	2.464 (1.458–4.164)	0.001	605.595 (367.291–998.515)	< 0.001	168.806 (66.267–430.011)	< 0.001
2018	1.556 (1.265–1.914)	< 0.001	0.798 (0.553–1.151)	0.227	10.126 (7.180–14.281)	< 0.001	3.825 (2.144–6.826)	< 0.001	1868.589 (866.222–4030.864)	< 0.001	640.428 (195.589–2096.988)	< 0.001
2019	2.471 (1.993–3.063)	< 0.001	1.213 (0.820–1.795)	0.333	63.924 (43.715–93.476)	< 0.001	10.154 (4.837–21.317)	< 0.001				
2020	4.648 (3.680–5.871)	< 0.001	2.110 (1.391–3.201)	< 0.001	153.950 (88.583–	< 0.001	70.312 (30.051–	< 0.001				

					267.552)		164.512)					
2021	9.719 (7.475–12.636)	< 0.001	5.442 (3.257–9.091)	< 0.001								
Stage												
Localized	Ref		Ref		Ref		Ref		Ref		Ref	
Regional	1.935 (1.768–2.117)	< 0.001	1.541 (1.348–1.761)	< 0.001	1.634 (1.452–1.839)	< 0.001	1.500 (1.250–1.799)	< 0.001	1.418 (1.222–1.646)	< 0.001	1.510 (1.185–1.925)	0.001
Distant	4.908 (4.401–5.474)	< 0.001	3.523 (2.904–4.275)	< 0.001	3.553 (3.005–4.200)	< 0.001	3.217 (2.383–4.344)	< 0.001	2.031 (1.548–2.665)	< 0.001	3.123 (1.912–5.099)	< 0.001
Unknown	1.697 (1.452–1.982)	< 0.001	1.640 (1.201–2.239)	0.002	1.312 (1.059–1.625)	0.013	1.647 (1.062–2.555)	0.026	1.189 (0.907–1.559)	0.210	1.318 (0.682–2.545)	0.412
Primary treatments												
Surgery alone	Ref				Ref				Ref			
Surgery + adjuvant radiotherapy	1.352 (1.211–1.510)	< 0.001			1.268 (1.098–1.464)	0.001			1.088 (0.908–1.305)	0.361		
Surgery + adjuvant chemotherapy	1.456 (1.197–1.770)	< 0.001			1.293 (1.001–1.670)	0.049			1.193 (0.874–1.627)	0.267		
Radiotherapy alone	3.007 (2.587–3.497)	< 0.001			2.546 (2.040–3.177)	< 0.001			2.133 (1.570–2.897)	< 0.001		
Radiotherapy + adjuvant chemotherapy	2.090 (1.848–2.364)	< 0.001			1.739 (1.471–2.056)	< 0.001			1.520 (1.232–1.875)	< 0.001		
Neoadjuvant chemotherapy + radiotherapy ± adjuvant chemotherapy	2.024 (1.793–2.286)	< 0.001			1.741 (1.470–2.062)	< 0.001			1.625 (1.301–2.029)	< 0.001		
Chemotherapy alone	3.013 (2.650–3.425)	< 0.001			2.343 (1.920–2.859)	< 0.001			1.745 (1.303–2.338)	< 0.001		
Surgery												
Total hysterectomy			Ref				Ref				Ref	
Radical hysterectomy			0.831 (0.636–1.086)	0.175			0.734 (0.482–1.117)	0.149			0.752 (0.328–1.724)	0.500
Trachelectomy (radical or simple)			1.167 (0.681–1.999)	0.575			0.760 (0.307–1.881)	0.553			0.742 (0.146–3.771)	0.719
Conization			0.810 (0.601–1.093)	0.168			0.797 (0.507–1.252)	0.325			1.003 (0.423–2.380)	0.995
Chemotherapy												
Platinum-based chemotherapy			Ref				Ref				Ref	
Other agents			1.163 (0.517–2.615)	0.715			1.005 (0.319–3.162)	0.994			0.624 (0.086–4.517)	0.641
Bevacizumab ± any agents			3.741 (3.025–4.628)	< 0.001			3.752 (2.646–5.321)	< 0.001			1.078 (0.333–3.491)	0.900
Postoperative VTE												
(-)	Ref		Ref		Ref		Ref		Ref		Ref	
(+)	1.286 (1.098–1.506)	0.002	1.322 (1.003–1.744)	0.048	1.389 (1.103–1.747)	0.005	1.671 (1.152–2.425)	0.007	2.099 (1.561–2.821)	< 0.001	2.306 (1.392–3.819)	0.001
Prophylactic anticoagulants												
(-)	Ref		Ref		Ref		Ref		Ref		Ref	
(+)	2.184 (2.034–2.345)	< 0.001	2.284 (1.998–2.611)	< 0.001	2.292 (2.075–2.532)	< 0.001	2.450 (2.034–2.952)	< 0.001	2.006 (1.766–2.278)	< 0.001	2.226 (1.738–2.850)	< 0.001

Duration of HRT (year)												
No use	Ref		Ref		Ref		Ref		Ref		Ref	
≤0.5	0.671 (0.605–0.745)	< 0.001	0.652 (0.557–0.763)	< 0.001	0.747 (0.652–0.857)	< 0.001	0.781 (0.635–0.960)	0.019	0.717 (0.601–0.856)	< 0.001	0.867 (0.658–1.142)	0.310
0.5-1	0.625 (0.543–0.719)	< 0.001	0.587 (0.476–0.724)	< 0.001	0.688 (0.575–0.823)	< 0.001	0.642 (0.490–0.842)	0.001	0.740 (0.589–0.930)	0.010	0.679 (0.472–0.978)	0.038
1-2					0.982 (0.737–1.308)	0.901	1.540 (0.888–2.671)	0.124	0.791 (0.533–1.174)	0.245	1.688 (0.736–3.871)	0.216
2-3					0.768 (0.481–1.226)	0.268	0.342 (0.048–2.458)	0.286	0.813 (0.475–1.390)	0.449	0.579 (0.079–4.254)	0.591
3-4									0.753 (0.281–2.022)	0.574	1.126 (0.150–8.434)	0.908
4-5									0.691 (0.298–1.603)	0.389	NE (NE) [a]	NE [a]

Abbreviations: CCI, Charlson comorbidity index; CI, confidence interval; HR, hazard ratio; HRT, hormone replacement therapy; OS, overall survival; SES, socioeconomic status; VTE, venous thromboembolism.

Multivariable analyses III and IV used the same predefined covariate sets as multivariable analyses I and II, respectively, with binary HRT use replaced by HRT duration before each landmark time point.

[a] HR, 95% CI, and p-value could not be reliably estimated for the 4–5 year HRT duration category due to adjustment-induced quasi-complete separation in the multivariable model. In univariable analysis, the corresponding HR was 3.659 (95% CI, 2.798–4.785; $p < 0.001$).
