# Supplementary material for: Impact of Post-Primary Treatment Hormone Replacement Therapy on Survival in Women with Cervical Cancer
Source: Cancers (Basel). 2026 Sep 2;18(17):2841. doi: 10.3390/cancers18172841 (PMC13564618; doi:10.3390/cancers18172841)
Supplement: Supplementary file 1 [file cancers-18-02841-s001.zip › Table S5 (Histology Cox).pdf]

Table S5. Sensitivity analysis using histology-stratified Cox regression.

	SCC				AC				ASC				NC			
	Multivariable analysis I		Multivariable analysis II		Multivariable analysis I		Multivariable analysis II		Multivariable analysis I		Multivariable analysis II		Multivariable analysis I		Multivariable analysis II	
Variables	HR (95% CI)	*p* value	HR (95% CI)	*p* value	HR (95% CI)	*p* value	HR (95% CI)	*p* value	HR (95% CI)	*p* value	HR (95% CI)	*p* value	HR (95% CI)	*p* value	HR (95% CI)	*p* value
Age (years)	0.983 (0.979–0.987)	< 0.001	0.985 (0.978–0.992)	< 0.001	1.004 (0.990–1.018)	0.592	1.014 (0.992–1.036)	0.227	0.970 (0.949–0.991)	0.005	0.976 (0.948–1.004)	0.094	1.012 (0.984–1.040)	0.421	1.023 (0.976–1.071)	0.344
SES																
Low SES	Ref		Ref		Ref		Ref		Ref		Ref		Ref		Ref	
Mid- or high-SES	0.841 (0.769–0.920)	< 0.001	0.859 (0.727–1.016)	0.075	1.053 (0.790–1.405)	0.725	0.711 (0.451–1.123)	0.144	0.591 (0.371–0.942)	0.027	0.630 (0.325–1.222)	0.172	0.431 (0.192–0.964)	0.041	0.225 (0.067–0.755)	0.016
CCI																
0	Ref		Ref		Ref		Ref		Ref		Ref		Ref		Ref	
1	0.891 (0.797–0.997)	0.044	0.926 (0.769–1.116)	0.419	1.026 (0.743–1.416)	0.876	1.147 (0.688–1.912)	0.600	0.730 (0.423–1.261)	0.260	1.489 (0.751–2.952)	0.254	1.162 (0.502–2.691)	0.726	1.065 (0.301–3.77)	0.923
2	0.926 (0.828–1.036)	0.179	0.824 (0.661–1.027)	0.085	0.778 (0.574–1.055)	0.106	0.830 (0.513–1.343)	0.447	0.841 (0.505–1.400)	0.506	0.465 (0.211–1.023)	0.057	1.300 (0.584–2.891)	0.520	1.706 (0.467–6.231)	0.419
3	0.849 (0.692–1.042)	0.117	0.722 (0.476–1.097)	0.127	1.216 (0.769–1.924)	0.403	1.352 (0.591–3.090)	0.475	0.625 (0.185–2.109)	0.449	0.687 (0.143–3.310)	0.640	0.297 (0.071–1.253)	0.098	0.125 (0.007–2.267)	0.160
≥4	1.231 (0.983–1.540)	0.070	1.623 (1.034–2.549)	0.035	0.840 (0.370–1.912)	0.678	0.850 (0.257–2.814)	0.790	0.559 (0.129–2.411)	0.435	0 (0– NE) [a]	0.984 [a]	2.468 (0.792–7.692)	0.119	0.662 (0.019–22.872)	0.820
Year of cancer diagnosis																
2007	Ref		Ref		Ref		Ref		Ref		Ref		Ref		Ref	
2008	1.183 (1.004–1.394)	0.044	1.067 (0.801–1.422)	0.656	1.633 (0.921–2.894)	0.093	0.745 (0.304–1.828)	0.520	0.789 (0.319–1.95)	0.607	0.626 (0.196–1.997)	0.429	0.903 (0.123–6.652)	0.920	1.281 (0.189–8.699)	0.800
2009	1.098 (0.917–1.314)	0.310	1.053 (0.769–1.443)	0.748	2.562 (1.498–4.379)	< 0.001	1.340 (0.637–2.817)	0.441	0.392 (0.149–1.034)	0.058	0.230 (0.065–0.814)	0.023	1.038 (0.117–9.197)	0.973	0.661 (0.084–5.201)	0.694
2010	1.171 (0.976–1.405)	0.089	1.010 (0.736–1.385)	0.952	1.871 (1.059–3.305)	0.031	0.805 (0.342–1.896)	0.620	0.673 (0.289–1.567)	0.358	0.255 (0.084–0.769)	0.015	0.930 (0.126–6.851)	0.943	1.987 (0.299–13.209)	0.478
2011	1.298 (1.080–1.559)	0.005	1.158 (0.841–1.594)	0.370	2.166 (1.211–3.872)	0.009	1.079 (0.481–2.425)	0.853	0.643 (0.272–1.522)	0.315	0.389 (0.119–1.272)	0.118	3.149 (0.400–24.794)	0.276	7.796 (0.776–78.314)	0.081
2012	1.264 (1.050–1.521)	0.013	1.033 (0.748–1.427)	0.843	2.022 (1.169–3.496)	0.012	1.591 (0.749–3.377)	0.227	0.881 (0.400–1.937)	0.752	0.465 (0.161–1.340)	0.156	0.961 (0.123–7.51)	0.970	0.552 (0.050–6.091)	0.627
2013	1.560 (1.304–1.866)	< 0.001	1.510 (1.116–2.043)	0.008	2.573 (1.464–4.522)	0.001	1.740 (0.762–3.970)	0.188	0.906 (0.405–2.028)	0.811	0.470 (0.168–1.316)	0.151	1.424 (0.176–11.483)	0.740	0.598 (0.047–7.527)	0.691
2014	1.310 (1.081–1.589)	0.006	0.928 (0.662–1.300)	0.663	1.625 (0.868–3.042)	0.129	0.707 (0.282–1.775)	0.461	1.137 (0.457–2.824)	0.783	0.294 (0.087–0.994)	0.049	0.988 (0.133–7.351)	0.990	0.662 (0.092–4.767)	0.682
2015	1.421 (1.172–	< 0.001	0.810 (0.571–	0.238	1.522 (0.797–	0.203	0.528 (0.204–	0.190	0.899 (0.384–	0.807	0.284 (0.091–	0.030	1.900 (0.167–	0.605	1.097 (0.104–	0.939

	1.722)		1.149)		2.907)		1.370)		2.106)		0.885)		21.675)		11.515)	
2016	1.391 (1.139–1.699)	0.001	0.782 (0.549–1.112)	0.171	1.607 (0.846–3.056)	0.148	0.281 (0.077–1.029)	0.055	1.071 (0.460–2.495)	0.873	0.480 (0.168–1.372)	0.171	1.039 (0.118–9.122)	0.973	4.355 (0.337–56.266)	0.260
2017	1.313 (1.063–1.622)	0.011	0.819 (0.561–1.197)	0.303	1.722 (0.848–3.5)	0.133	0.786 (0.245–2.522)	0.686	0.921 (0.352–2.41)	0.867	0.33 (0.077–1.421)	0.137	0.627 (0.073–5.392)	0.670	0.884 (0.074–10.561)	0.922
2018	1.329 (1.068–1.653)	0.011	0.796 (0.532–1.191)	0.266	1.783 (0.894–3.555)	0.101	0.437 (0.138–1.388)	0.160	0.937 (0.356–2.466)	0.895	0.249 (0.064–0.966)	0.044	2.242 (0.276–18.186)	0.450	1.215 (0.056–26.525)	0.901
2019	2.086 (1.666–2.613)	< 0.001	1.222 (0.797–1.873)	0.358	2.705 (1.346–5.435)	0.005	0.611 (0.157–2.372)	0.477	2.166 (0.757–6.199)	0.150	0.861 (0.200–3.711)	0.841	1.455 (0.205–10.324)	0.708	2.833 (0.310–25.88)	0.356
2020	3.169 (2.471–4.065)	< 0.001	1.646 (0.987–2.746)	0.056	5.692 (2.647–12.24)	< 0.001	1.335 (0.44–4.051)	0.610	1.543 (0.447–5.321)	0.492	0.458 (0.09–2.325)	0.346	2.362 (0.297–18.801)	0.417	3.325 (0.404–27.386)	0.264
2021	6.729 (5.087–8.901)	< 0.001	7.54 (4.049–14.038)	< 0.001	5.496 (2.735–11.046)	< 0.001	1.587 (0.447–5.632)	0.475	8.364 (1.625–43.041)	0.011	0.737 (0.055–9.79)	0.817	4.078 (0.430–38.699)	0.221	NE [b]	NE [b]
Stage																
Localized	Ref		Ref		Ref		Ref		Ref		Ref		Ref		Ref	
Regional	1.872 (1.698–2.064)	< 0.001	1.483 (1.275–1.724)	< 0.001	2.562 (1.925–3.411)	< 0.001	2.227 (1.501–3.302)		2.574 (1.686–3.931)	< 0.001	1.377 (0.799–2.374)	0.249	3.770 (1.884–7.545)	< 0.001	8.852 (3.071–25.513)	< 0.001
Distant	4.739 (4.220–5.321)	< 0.001	3.487 (2.818–4.315)	< 0.001	6.886 (4.891–9.696)	< 0.001	7.737 (4.317–13.867)		8.345 (4.315–16.138)	< 0.001	2.030 (0.621–6.633)	0.241	11.701 (4.722–28.994)	< 0.001	12.077 (2.506–58.198)	0.002
Unknown	1.632 (1.381–1.928)	< 0.001	1.48 (1.031–2.125)	0.034	1.990 (1.273–3.11)	0.003	1.747 (0.807–3.781)	0.157	1.483 (0.498–4.418)	0.480	8.525 (0.848–85.661)	0.069	2.696 (0.683–10.638)	0.157	2.957 (0.185–47.388)	0.444
Primary treatments																
Surgery alone	Ref				Ref				Ref				Ref			
Surgery + adjuvant radiotherapy	1.349 (1.194–1.525)	< 0.001			1.850 (1.344–2.546)	< 0.001			0.757 (0.464–1.235)	0.265			1.864 (0.569–6.099)	0.304		
Surgery + adjuvant chemotherapy	1.498 (1.209–1.856)	< 0.001			1.535 (0.868–2.716)	0.141			1.400 (0.585–3.351)	0.450			1.970 (0.316–12.294)	0.468		
Radiotherapy alone	3.302 (2.811–3.878)	< 0.001			3.571 (2.222–5.739)	< 0.001			1.833 (0.509–6.597)	0.354			1.949 (0.430–8.823)	0.387		
Radiotherapy + adjuvant chemotherapy	2.329 (2.041–2.658)	< 0.001			2.614 (1.756–3.890)	< 0.001			2.360 (1.219–4.567)	0.011			0.356 (0.053–2.403)	0.289		
Neoadjuvant chemotherapy + radiotherapy ± adjuvant chemotherapy	2.218 (1.947–2.527)	< 0.001			2.470 (1.618–3.770)	< 0.001			1.450 (0.755–2.786)	0.265			4.758 (1.054–21.480)	0.043		
Chemotherapy alone	3.527 (3.076–4.044)	< 0.001			3.421 (2.255–5.189)	< 0.001			2.583 (1.32–5.055)	0.006			2.023 (0.532–7.686)	0.301		
Surgery																
Total hysterectomy			Ref				Ref				Ref				Ref	
Radical hysterectomy			0.783 (0.578–	0.113			0.724 (0.334–	0.411			0.367 (0.122–	0.075			31.719 (0.105–	0.235

			1.060)				1.565)				1.107)				9555.472)	
Trachelectomy (radical or simple)			1.356 (0.759–2.424)	0.303			0.587 (0.068–5.038)	0.627			0.222 (0.022–2.186)	0.197			NE [c]	NE [c]
Conization			0.796 (0.57–1.111)	0.179			1.145 (0.485–2.702)	0.757			0.136 (0.030–0.607)	0.009			254.347 (0.658–98302.19)	0.068
Chemotherapy																
Platinum-based chemotherapy			Ref				Ref				Ref				Ref	
Other agents			0.637 (0.203–1.992)	0.438			3.951 (0.874–17.853)	0.074			NE [c]	NE [c]			1.230 (0.018–82.178)	0.923
Bevacizumab ± any agents			3.93 (3.11–4.965)	< 0.001			4.437 (2.005–9.820)	< 0.001			2.819 (1.170–6.79)	0.021			10.162 (1.736–59.483)	0.010
Postoperative VTE																
(-)	Ref		Ref		Ref		Ref		Ref		Ref		Ref		Ref	
(+)	1.194 (1.002–1.422)	0.047	1.278 (0.926–1.763)	0.136	0.899 (0.563–1.436)	0.656	0.862 (0.413–1.800)	0.693	2.629 (1.336–5.172)	0.005	2.676 (0.974–7.355)	0.056	0 (0-NE) [d]	0.985 [c]	0.144 (0–486.048)	0.641
Prophylactic anticoagulants																
(-)	Ref		Ref		Ref		Ref		Ref		Ref		Ref		Ref	
(+)	2.130 (1.976–2.297)	< 0.001	2.299 (1.98–2.670)	< 0.001	1.958 (1.559–2.46)	< 0.001	1.816 (1.232–2.677)	0.003	3.346 (2.214–5.056)	< 0.001	3.913 (2.092–7.317)	< 0.001	1.197 (0.661–2.166)	0.553	1.564 (0.583–4.192)	0.374
HRT																
(-)	Ref		Ref		Ref		Ref		Ref		Ref		Ref		Ref	
(+)	0.522 (0.477–0.571)	< 0.001	0.571 (0.493–0.662)	< 0.001	0.517 (0.392–0.683)	< 0.001	0.677 (0.447–1.024)	0.065	0.626 (0.414–0.947)	0.027	0.605 (0.349–1.049)	0.073	0.734 (0.370–1.457)	0.377	2.966 (0.989–8.896)	0.052

Abbreviations: AC, cervical adenocarcinoma; ASC, adenosquamous carcinoma; CCI, Charlson comorbidity index; CI, confidence interval; HR, hazard ratio; HRT, hormone replacement therapy; NC, neuroendocrine carcinoma; NE: not estimable; SES, socioeconomic status; SCC, squamous cell carcinoma; VTE, venous thromboembolism.

Multivariable analyses I and II were adjusted using the same predefined covariate sets as those used throughout the study.

[a] The upper limit of the 95% CI is denoted "NE" because it was not estimable. The hazard ratio, 95% CI, and *p* value for CCI ≥4 in the multivariable model should be interpreted with caution due to quasi-complete separation resulting from sparse events within this subgroup after adjustment for other covariates in the model. In the univariable model, the HR for CCI ≥4 was 0.465 (95% CI, 0.115–1.884; $p = 0.283$).

[b] The hazard ratio, 95% CI, and p-value for YEAR=2021 in the multivariable model could not be reliably estimated due to model non-convergence (likely reflecting separation in the opposite direction from that observed for CCI ≥4 and VTE, i.e., divergence of the estimate toward an extremely large value). The apparent $p < 0.001$ is a numerical artifact and does not indicate a statistically meaningful effect. In the univariable model, the HR for YEAR=2021 was 12.42 (95% CI, 1.790–86.18; $p = 0.011$).

[c] No patients in the ASC group received other agents as chemotherapy, and no patients in the NC group underwent trachelectomy (as surgery); therefore, the hazard ratio, 95% CI, and *p* value could not be estimated for these categories.

[d] The hazard ratio, 95% CI, and p-value for VTE could not be reliably estimated because no events occurred among patients with VTE, resulting in complete separation. The upper limit of the 95% CI is denoted "NE" because it was not estimable. A similar result was observed in the univariable analysis (HR, 0.000; 95% CI, 0.000–NE; $p = 0.985$).
